# ADViSELipidomics: a workflow for analyzing lipidomics data

**DOI:** 10.1093/bioinformatics/btac706

**Published:** 2022-10-29

**Authors:** Eugenio Del Prete, Ana Margarida Campos, Fabio Della Rocca, Carmela Gallo, Angelo Fontana, Genoveffa Nuzzo, Claudia Angelini

**Affiliations:** Institute for Calculus Applications ‘M. Picone’, CNR, 80131 Naples, Italy; Institute of Biomolecular Chemistry, CNR, 80078 Naples, Italy; Institute for Calculus Applications ‘M. Picone’, CNR, 80131 Naples, Italy; Institute of Biomolecular Chemistry, CNR, 80078 Naples, Italy; Institute of Biomolecular Chemistry, CNR, 80078 Naples, Italy; Department of Biology, University of Naples “Federico II”, 80126 Naples, Italy; Institute of Biomolecular Chemistry, CNR, 80078 Naples, Italy; Institute for Calculus Applications ‘M. Picone’, CNR, 80131 Naples, Italy

## Abstract

**Summary:**

ADViSELipidomics is a novel Shiny app for preprocessing, analyzing and visualizing lipidomics data. It handles the outputs from LipidSearch and LIQUID for lipid identification and quantification and the data from the Metabolomics Workbench. ADViSELipidomics extracts information by parsing lipid species (using LIPID MAPS classification) and, together with information available on the samples, performs several exploratory and statistical analyses. When the experiment includes internal lipid standards, ADViSELipidomics can normalize the data matrix, providing normalized concentration values per lipids and samples. Moreover, it identifies differentially abundant lipids in simple and complex experimental designs, dealing with batch effect correction. Finally, ADViSELipidomics has a user-friendly graphical user interface and supports an extensive series of interactive graphics.

**Availability and implementation:**

ADViSELipidomics is freely available at https://github.com/ShinyFabio/ADViSELipidomics

**Supplementary information:**

Supplementary data are available at *Bioinformatics* online.

## 1 Introduction

Lipids are biologically active and ubiquitous metabolites involved in cell structure, energy storage and metabolism, signaling and other different functions (Kyle *et al.*, 2021). Alterations in lipid homeostasis are associated with many disturbances (e.g. protein dysfunction and insulin resistance) or pathologies (e.g. metabolic syndrome and obesity). Lipids play multiple roles due to the numerous lipid classes and species, each with a particular structure. Lipidomics consists of methodologies and techniques for identifying and quantifying the lipid pool (lipidome) in a specific state or condition. In recent years, increasingly developed technologies, such as liquid chromatography integrated with tandem mass spectrometry (LC-MS/MS), allowed identifying lipids as disease biomarkers (Han, 2016). Due to the ever-increasing interest in lipids, Lipid Metabolites and Pathways Strategy (LIPID MAPS) web portal was launched in 2003 to standardize lipid classification and provide a curated database (Liebisch *et al.*, 2020). Moreover, the SwissLipids portal constitutes a knowledge resource for lipids and their biology (Aimo *et al.*, 2015) connected with LIPID MAPS. Furthermore, Metabolomics Workbench is a repository for metabolomics data and metadata that provides analysis tools and includes several experiments related to lipidomics (Sud *et al.*, 2016).

Considering the increasing availability of online repositories and the complexity and amount of data generated by LC-MS/MS approaches, computational tools have become essential to handling the huge amount of obtained data. Consequently, researchers developed many tools to process raw data produced by different instruments, primarily focusing on raw data processing. LipidBlast (Kind *et al.*, 2013) and LipidMatch (Koelmel *et al.*, 2017) perform lipid annotation from MS/MS data. In a similar way, LipidMS (Alcoriza-Balaguer *et al.*, 2019) is an R package for lipid annotation from both MS and MS/MS data, followed by initial data processing. LipidSearch is a well-known commercial software for preprocessing both LC-MS and LC-MS/MS data, providing accurate peak-picking identification and integration, and visualization of results. LipidFinder is an open-source Python workflow that combines several different databases to obtain the putative identification of lipids by LC-MS (Alvarez-Jarreta *et al.*, 2021). Lipid Quantification and Identification (LIQUID) is a C# open-source software that allows semi-automated processing of data against a customizable target library, and visualization of LC-MS/MS lipidomic data (Kyle *et al.*, 2017). After identifying and quantifying lipids, data should be preprocessed and analyzed using advanced statistical methods. Within the project ADViSE (Antitumor Drugs and Vaccines from the Sea), we developed ADViSELipidomics, a comprehensive R Shiny user-friendly app for an interactive preprocessing, analysis and visualization of lipidome annotated data.

### 1.1 Other tools and ADViSELipidomics

As mentioned in the Section 1, several bioinformatics tools for MS-based lipidomic data analysis have been developed in recent years. Some tools are focused on lipid identification and quantification, others on statistical analyses (to find lipid abundance differences associated with experimental conditions or diseases), and only a few provide a comprehensive platform. ADViSELipidomics falls in the second group of tools. Among the tools specifically devoted to lipidomics statistical analysis, LipidSig (Lin *et al.*, 2021) and lipidR (Mohamed *et al.*, 2020) provide several useful functionalities as command-line or web-server. Both allow uploading lipidomics datasets, performing statistical analysis (limited to simple experimental designs), and data exploration. Instead, Lipostar (Goracci *et al.*, 2017) and MS-DIAL (Tsugawa *et al.*, 2020) are more comprehensive tools capable of processing and aligning raw data, lipid identification and performing multivariate analysis.

Compared to the above-mentioned tools, ADViSELipidomics provides some overlapping functionalities and specific features that make it an alternative and complementary tool for accurately analyzing lipidomic data. More in detail, ADViSELipidomics allows the upload of lipid data from different sources. Moreover, it provides the possibility to use internal standard (IS) and obtain calibration curves, then it performs normalized absolute quantification of individual lipid species in each sample on data derived from LipidSearch. Indeed, the possibility of dealing with IS is one of the most remarkable advantages of ADViSELipidomics. To our knowledge, ADViSELipidomics is the only software that grants the use of calibration curves during data analysis, to convert peak area into normalized concentrations, considering the linearity range of the instrument response. ADViSELipidomics computes the percentage of recovery for each sample, providing a more accurate comparison among samples, as demonstrated by the results of our case study one in the Supplementary Material. Furthermore, ADViSELipidomics provides a comprehensive system of data filtering to minimize the presence of false positives, considering several features, such as the retention time of the lipid classes, the length and the number of double bonds present in the fatty acyl chains, checking for fatty acyl chains with an odd number of carbons. Finally, ADViSELipidomics also improves other existing methods in terms of statistical analysis, allowing the analysis of complex experimental designs and removing batch effects, which are rarely supported in other tools. Moreover, similar to other tools, ADViSELipidomics allows carrying out data exploration with several multivariate approaches.

## 2 Implementation

The logical workflow implemented in ADViSELipidomics consists of the following tasks:

- **Data import**. After selecting the type of input data to analyze (LipidSearch, LIQUID, Metabolomics Workbench, Excel file or *SummarizedExperiment* object), the software requests to upload a series of mandatory or optional files. Mandatory files are the data files and a target file containing information on the samples. Optional files are calibration files and an internal reference file. Then, it imports the data, pre-filters them based on features of interest, and parses lipids to obtain a deeper stratification of their characteristics (class, chain length and double bonds).
- **Data preprocessing**. ADViSELipidomics performs a filtering step based on boundaries in the internal reference file, focused on double bonds, carbon atoms and lipid duplicates. This step reduces false positive lipid identification. Then, if internal standard lipids are available, it copes with their presence and calculates recovery percentage to apply to each lipid to obtain an abundance matrix as normalized corrected concentrations. This filtering and preprocessing step constitutes one of the most innovative features that distinguish ADViSELipidomics from other existing methods. After this step, it allows filtering samples or lipids, and using algorithms to impute missing values. Information on samples, lipids and abundances are stored in a *SummarizedExperiment* R object that can be explored.
- **Exploratory analysis**. The software allows performing different interactive plots on lipids and samples, e.g. density plots, spider plots, scatter plots and heatmaps. Furthermore, there are two dimension reduction methods, i.e. Principal Component Analysis (unsupervised) and Partial Least Square—Discriminant Analysis (supervised) and several clustering algorithms. All plots are interactive and highly customizable.
- **Statistical analysis**. ADViSELipidomics allows comparing lipid abundance between several groups of interest, e.g. treatment versus control, by choosing the experimental groups among the metadata given in the target file. For this purpose, it supports the user in defining the experimental design (simple and complex), handling or removing batch effects, and choosing the comparison. Then, it performs the differential analysis, depicting the results with MA plots and Volcano plots. Finally, from the results of the differential analysis, it evaluates different lipid sets based on the lipid parsing and identifies which are statistically enriched.

**Fig. 1.**
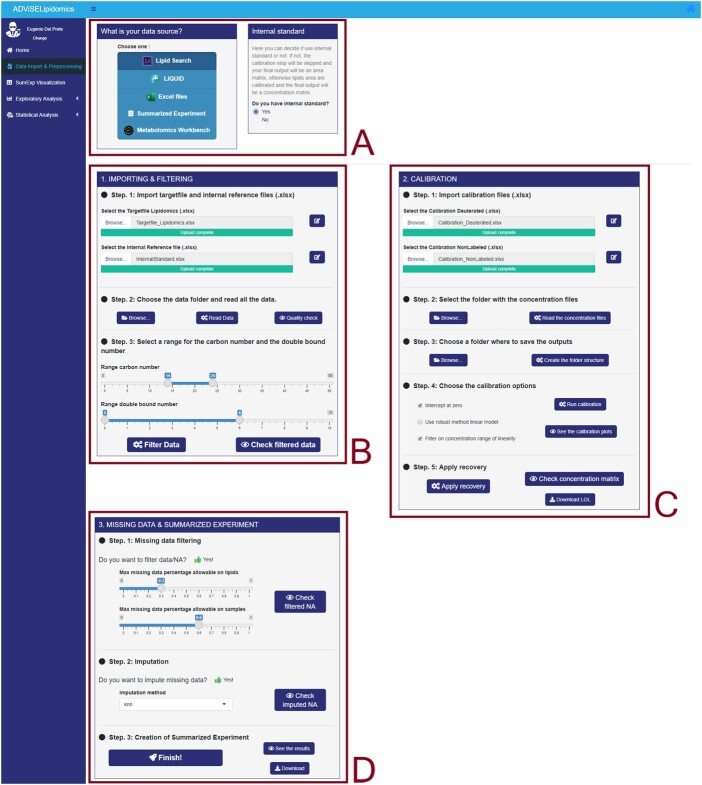
ADViSELipidomics interface. The example shows selection (**A**), import and filtering (**B**), calibration (**C**) of data, imputation of missing data and creation of Summarized Experiment R object (**D**)

ADViSELipidomics is a stand-alone application implemented using the R language (R > 4.0) and the shiny libraries. We tested on several operating systems (Windows, macOS and Linux). Figure 1 shows a screenshot of the interface. Technical details are described in the Supplementary Material and the user manual.

## 3 Results

To show the use and the capabilities of ADViSELipidomics, we analyzed two case studies: (i) primary human monocytes compared to human immature dendritic cells (iDCs) derived from primary human monocytes, with a novel UHPLC-HRESIMS-MS/MS method, which uses internal lipid standards to normalize raw data according to the instrument response and the efficiency of lipid recovery during lipid extraction, providing an absolute quantification per lipid species; (ii) lipid profile data of serum samples of healthy versus diabetic individuals, available from Metabolomic Workbench repository. Note that the first case study not only illustrates the performance of ADViSELipidomics using real data but also clearly shows the advantage of our UHPLC-HRESIMS-MS/MS method and the use of internal standards to obtain normalized absolute lipid quantification.

Further details on functionalities, input/output data, parameters, and results are in the Supplementary Material.

## 4 Conclusions

ADViSELipidomics is a Shiny app written in R language, with an easy-to-use and point-and-click graphical user interface, powered with interactive plots. It provides a comprehensive open-source tool for analyzing lipidomics experiments processing different data files, e.g. obtained from peak integration and quantification tools. Remarkably, when combined with our new UHPLC-HRESIMS-MS/MS method, ADViSELipidomics can take advantage of internal lipid standard information (if available in the experiment to analyze) to get an accurate quantification in terms of concentration. Furthermore, thanks to the modular structure, ADViSELipidomics allows the preprocessing, filtering, exploration and analysis of lipidomics data and can be easily improved by adding new functionalities.

## Funding

The work was supported by ‘Antitumor Drugs and Vaccines from the Sea (ADViSE)’ project [CUP B43D18000240007–SURF 17061BP000000011] funded by POR Campania FESR 2014-2020.

*Conflict of Interest*: none declared.

## Supplementary Material

btac706_Supplementary_Data

## Data Availability

The data underlying this article are available at the link in the Section 'Project Links' in the Supplementary Material for the first case study, downloadable from the Shiny app or accessed directly via it's Project https://doi.org/10.21228/M8J60X at the Metabolomic Workbench repository (Project ID PR000445, Study ID ST000608) for the second case study.
