# Supplementary material for: ADViSELipidomics: a workflow for analyzing lipidomics data
Source: Bioinformatics. 2022 Oct 29;38(24):5460–2. doi: 10.1093/bioinformatics/btac706 (PMC9750127; doi:10.1093/bioinformatics/btac706)
Supplement: btac706_Supplementary_Data [file btac706_supplementary_data.docx]

**Supplementary Material to:**

**“ADViSELipidomics: a workflow for analyzing lipidomics data”**

**ADViSELipidomics Pipeline, Case Studies, Results, Analytic Details**

Eugenio Del Prete, Ana Margarida Campos, Fabio Della Rocca,

Carmela Gallo, Angelo Fontana, Genoveffa Nuzzo, and Claudia Angelini

**Table of Contents**

Abbreviations

1. ADViSELipidomics Details
   1. Workflow
      1. Data Import
      2. Data Preprocessing
      3. Exploratory Analysis
      4. Statistical Analysis
   2. Shiny App Interface
   3. Type of Data
      1. Input
      2. Output
   4. R packages
   5. Project Links
2. Case Studies
   1. Case Study One
   2. Case Study Two
3. Results

3.1 Improved LC-MS/MS Method

3.2 Case Study One

3.2.1 Data Analysis using Internal Standards

3.2.2 Data Analysis without using Internal Standards

3.3 Case Study Two

1. Analytic Details
   1. Method Validation and Validation Study
      1. Chemicals and Reagents
      2. Standard Mixture
      3. Selectivity
      4. External Calibration Curves
      5. Lower Limit of Quantification
      6. Carry-over Effect
      7. Matrix Effect
      8. Accuracy and Precision
   2. Case Study One
      1. Human Monocyte Isolation
      2. Immature Dendritic Cells Culture Conditions
      3. Lipid Extraction
      4. UHPLC-HRESIMS-MS/MS
2. Acknowledgements
3. Funding
4. References

**Abbreviations**

Cer Ceramide

ChE Cholesteryl Ester

CLARA CLustering LArge Applications

CV Coefficient of Variation

DBS Dried Blood Spots

DC Dendritic Cell

DG DiacylGlycerol

DGDG DiGalactosylDiacylGlycerol

EMA European Medicines Agency

FDA Food and Drug Administration

GM-CSF Granulocyte Macrophage-Colony Stimulating Factor

GUI Graphical User Interface

hDC human Dendritic Cell

hMo human Monocyte

HPLC High-Performance Liquid Chromatography

HRESI High Resolution ElectroSpray Ionization

iDC immature Dendritic Cell

IRMI Iterative Robust Model-based Imputation

IS Internal (lipid) Standard

KNN K-Nearest Neighbor

LC-MS Liquid Chromatography Mass Spectrometry

LLOQ Lowest Limit Of Quantification

logFC logarithmic Fold Change

LPC Lyso - (glycero) PhosphatidylCholine

LPE Lyso - (glycero) PhosphatidylEthanolamine

LPG Lyso - (glycero) PhosphatidylGlycerol

LPS LipoPolySaccharide

MF Matrix Factor

MGDG MonoGalactosylDiacylGlycerols

MS Mass Spectrometry

MTBE Methyl Tert-Butyl Ether

NA Not Available

PAM Partition Around Medoids

PBMC Peripheral Blood Mononuclear Cell

PC (glycero) PhosphatidylCholine

PCA Principal Component Analysis

PE (glycero) PhosphatidylEthanolamine

PG (glycero) PhosphatidylGlycerol

PLS-DA Partial Least Square - Discriminant Analysis

ppm parts per million

PS (glycero) PhosphatidylSerine

QC Quality Control

RT Retention Time

SE SummarizedExperiment

SM SphingoMyelin

SQDG SulfoQuinovosylDiacylGlycerol

TG TriacylGlicerol

UHPLC Ultra High-Performance Liquid Chromatography

**1. ADViSELipidomics Details**

***1.1 Workflow***

ADViSELipidomics is a Shiny app with a complete workflow for preprocessing and analyzing lipidomics data from different sources. The user can upload the data files together with lipid and sample details, select filters and statistical methods to apply to the dataset, and obtain the results as tables and interactive plots. ADViSELipidomics consists of four main logic tasks: Data Import, Data Preprocessing, Exploratory Analysis, and Statistical Analysis. Each task consists of different modules (described in the following subsections). Figure 1 shows the ADViSELipidomics workflow.


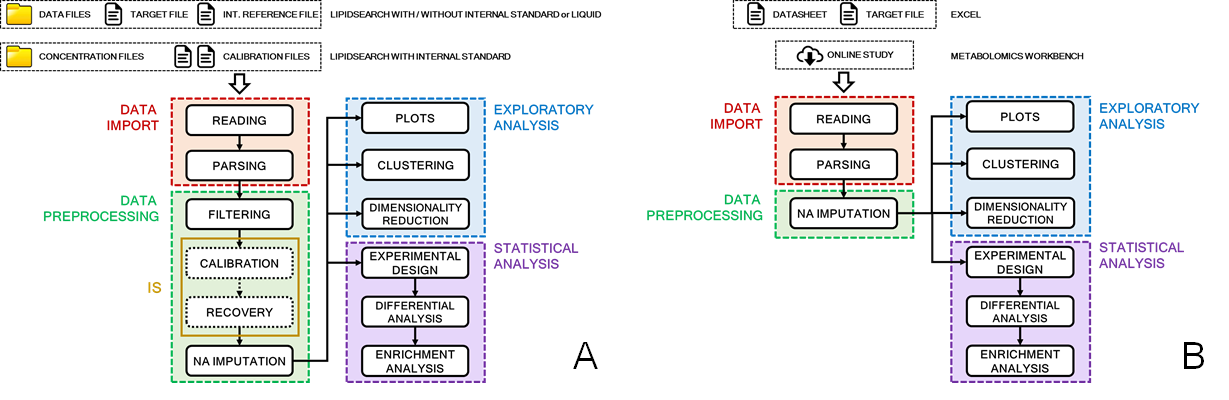


**Figure 1. ADViSELipidomics logical workflow.** Panel A (on the left) shows the workflow for LipidSearch and LIQUID input. Panel B (on the right) shows the workflow for Excel and Metabolomics Workbench input. The tasks are enlightened with different colors and consist of several modules. Calibration and Recovery modules are executed only when the experiment contains Internal Standard (IS).

1.1.1 Data Import

The Data Import task includes two modules: Reading and Parsing.

The **Reading module** allows the user to import different types of data files:

1. *LipidSearch or LIQUID*. ADViSELipidomics deals with the data files containing information on chromatographic peak area or peak intensity per lipid, obtained as output from external software for identifying and quantifying lipids (i.e., it currently supports the output formats from LipidSearch or LIQUID). Moreover, it also requires a Target File with details on samples (such as treatments, cell types, or biological replicates) and an Internal Reference File with information for the filtering step. ADViSELipidomics shows a quality plot based on the sum of chromatographic peak area per sample (or replicate). In the case of LipidSearch output associated with Internal (lipid) Standards (ISs), ADViSELipidomics also requires all the Calibration Files to construct the calibration curves. Moreover, ADViSELipidomics also allows editing the Target File, the Internal Reference File, and the Calibration Files, e.g., pre-filtering column variables or removing samples/lipids;
2. *Metabolomics Workbench.* ADViSELipidomics can download in real-time suitable selected lipidomic experiments from the online repository;
3. *Excel*. The user can upload two Excel files, the data matrix, and the Target File;
4. *SummarizedExperiment.* The user can upload a SummarizedExperiment R object (SE) with several types of information (data matrix, information on lipids, information on samples, metadata if available).

We underline that the quantification of lipids can vary for each experiment, and the user can choose the most appropriate. Differences are due to the quantification software and the presence of ISs. In ADViSELipidomics, the lipid quantification can be concentration, chromatographic peak area, chromatographic peak intensity, or other suitable measures. From this point in the manuscript, we will use the generic term ‘abundance’ (if not expressly reported differently).

Detailed information about ADViSELipidomics input files is given in Section 1.3 Data Type and in the online user’s manual.

The **Parsing module** extracts the information of lipids following nomenclature and classification reported in LIPID MAPS online resource. An example is depicted in Figure 2, where the lipid codification provides several features: class lipid from the head (phosphocholine, PC), length of the first tail (18 carbon atoms), number of double bonds in the first tail (0), length of the second tail (18 carbon atoms), number of double bonds in the second tail (1), position of the double bonds in the second tail (11Z). Other features can be present in the codification, and we parse all of them in tabular form, suitable for use in the following modules.

Detailed information about the lipid nomenclature supported in ADViSELipidomics is given in the online user manual.


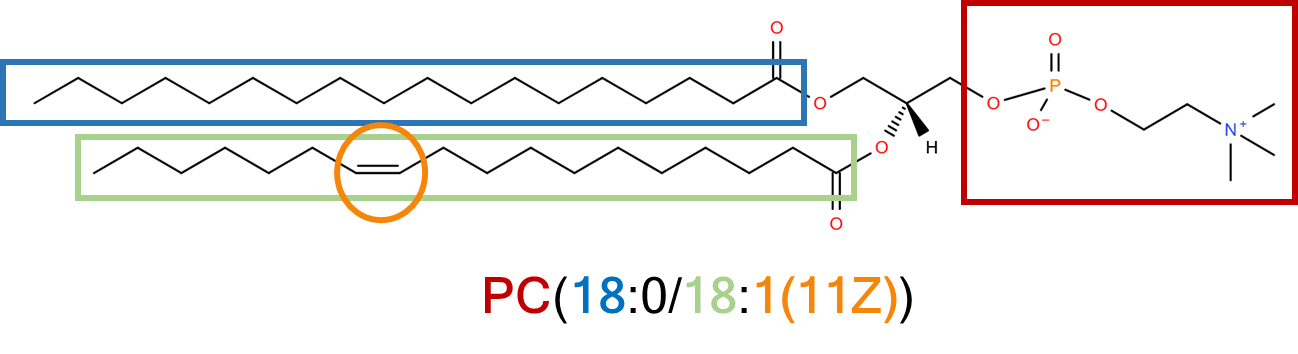


**Figure 2. Example of nomenclature and classification from LIPID MAPS.** The lipid is a glycerophosphatidylcholine (1-octadecanoyl-2-(11Z-octadecenoyl)-sn-glycero-3-phosphocholine, code LMGP01010750). The polar head in red defines the class, the hydrophobic tails are colored in blue and green, the presence and position of a double bond is colored in yellow.

1.1.2 Data Preprocessing

The Data Preprocessing task includes four modules: Filtering, Calibration, Recovery, and Missing Data Imputation. One of the most remarkable novelties in ADViSELipidomics lies in Calibration and Recovery modules, which introduce a novel approach to process lipid quantification with ISs to obtain a matrix with normalized corrected concentration values for each lipid and sample. ADViSELipidomics executes these two modules only when the user uploads experimental data files from LipidSearch and uses ISs (as in Section 3.1). Note that the calibration and recovery steps, that provide normalized absolute lipid quantification, constitute the main novelty of ADViSELipomics compared to other existing tools.

The **Filtering module** allows filtering non-informative lipids based on the upper/lower bounds (i.e., range) reported in the Internal Reference File and coping with multiple peak areas for one lipid. ADViSELipidomics applies filters per lipid on: retention time (RT) in the range, number of carbon atoms in the range, even number of carbon atoms, number of double bonds in the range, duplicated lipids. The same m/z values for lipid peaks identify duplicated lipids, and only the lipids with the maximum peak area are conserved.

The **Calibration module** creates the calibration curves and the calibration matrix. It uses the ISs reported in the Internal Reference file, and the correspondence between the Concentration Files and the lipid classes declared in the Calibration File. This module extracts the relationships between peaks area and concentration values for each IS, constructing the calibration curves with a linear model and plotting them. The linear regression model can be classical or robust (see Section 1.4), with zero or non-zero intercept. Finally, the calibration matrix resumes all the points from the calibration curves. After the calibration process, ADViSELipidomics stores slope and intercept values for the recovery module.

The **Recovery module** performs the computation and the application of the recovery percentage on the concentration values for each lipid, considering the ISs as lipid class reference. This correction provides normalized corrected concentration values for the lipids, that are a more accurate quantification measure than peak area or peak intensity. ADViSELipidomics uses the following approach: first, it estimates the recovery percentage from the IS using Eq (1); then, it predicts the corrected concentration using the estimated recovery percentage with Eq (2); finally, the corrected concentration is normalized as in Eq (3):

$$RP=\frac{PA-q}{m} \frac{100}{NSC} \left( 1 \right)$$

$$CC=\frac{PA-q}{m} \frac{100}{RP} \left( 2 \right)$$

$$NCC= \frac{CC}{NF} \left( 3 \right)$$

where $RP$ recovery percentage, $PA$ lipid peak area, $q$ intercept of the calibration curve, $m$ slope of the calibration curve, $NSC$ nominal standard concentration, $CC$ corrected concentration, *NCC* normalized corrected concentration, *NF* normalization scale factor. After the recovery process, ADViSELipidomics stores the concentration matrix with the normalized corrected concentrations. Lipid quantification as normalized corrected concentrations is possible only when using ISs. Normalized corrected concentrations are a more accurate and reproducible measure of quantification. To the best of our knowledge, ADViSELipidomics is the first software to provide these steps in an automatized workflow.

The **Missing data Imputation module** copes with the absence of some values in the abundance matrix. First, ADViSELipidomics computes the percentage of missing data values (denoted NA, Not Available) for each lipid (matrix rows) and each sample (matrix columns). Second, it allows retaining only lipids and/or samples with a percentage of missingness below user-selected thresholds. Finally, the lists of the filtered lipids/samples are stored with the results. After that, ADViSELipidomics shows the missing data distributions and allows the user to use two kinds of imputation methods:

- *Not Model-Based*. This set of algorithms does not consider the existence of an intrinsic model in the abundance matrix. The available algorithms are: a) median, b) mean, c) k-Nearest Neighbor (KNN). We suggest these methods with multiple replicates per sample;
- *Model-Based*. The Iterative Robust Model-based Imputation (IRMI) algorithm considers one variable (sample) as a response variable and the other variables as regressors. Hence, it imputes values in the response given the information on the regressors. The procedure is repeated iteratively for all the samples. We suggest this method without multiple replicates per sample.

It is always a good suggestion to execute lipid and sample filtering, then missing data imputation. In case of skipping this step and keeping missing data in the dataset, a warning message is shown to explain that some technical problems might arise in subsequent analysis. The effective impact in the subsequent analysis will depend on the amount of missingness in the data.

At the end of the Missing Data Imputation module, ADViSELipidomics creates a SE object suitable for the following steps, incorporating the abundance matrix, the samples information from the target file, the lipids information from the Parsing module, and other metadata if available. When multiple (technical) replicates per condition are present in the uploaded experiment, ADViSELipidomics creates two SE objects: the first where each replicate represents a sample, and the second where the technical replicates are averaged.

1.1.3 Exploratory Analysis

The Exploratory Analysis task includes three modules: Plots, Clustering, and Dimension Reduction.

The **Plots module** allows the user to create different types of plots explore the data from lipid and/or sample points of view:

- *Lipid plots*. It is possible to 1) represent the lipid class distribution (counts of lipids per class) with a pie chart, boxplot, and spider plot; 2) visualize the percentage of lipid class for each sample using a barplot, 3) compare the lipid species abundance for each condition; 4) inspect the abundance of a lipid, selected by the user, in relationship with a feature from the Target file (e.g., treatment or condition) using boxplots;
- *Scatterplots*. It is possible to visualize the lipid abundance between two samples;
- *Heatmap*. It provides a highly customizable heatmap to show possible clusters among lipids or samples. The user can select many parameters: a) row annotation with the feature from the target file, b) column annotation with the information from lipids parsing, c) dendrograms for lipids and/or samples, d) distance function (Euclidean, maximum, Canberra), e) clustering method (complete, average, median, Ward); f) number of clusters for lipids and/or samples. The user can select an area in the overall heatmap and have a detailed zoom of the area itself, with associated information;
- *Quality plots*. It provides different plots (barplot, boxplot, density plot) to show the total amount of abundance (logarithmic scale) *per* sample, considering a feature from the target file as reference in order to identify possible unexpected behavior among samples or replicates for the same sample.

The **Clustering module** allows the user to cluster the data by lipids or samples. The user can choose the number of clusters and the clustering method among the following algorithms: hierarchical clustering (using single, complete, Ward as linkage function) or partitioning clustering (k-means, PAM, CLARA). Additional plots, such as the silhouette plot, can suggest the number of clusters to use.

The **Dimensionality Reduction module** allows the user to choose between unsupervised and supervised approaches to represent the data in a two or three-dimensional space:

- *Unsupervised approach*. ADViSELipidomics computes the Principal Component Analysis (PCA), showing the results with different plots: a) 2D plot, b) biplot, c) scree plot, d) loadings plot, e) 3D plot. The user can highlight the features from the target file with different colors and select the number of components to use for the loading plots;
- *Supervised approach*. ADViSELipidomics computes the Partial Least Square-Discriminant Analysis in classical (PLS-DA) and sparse (sPLS-DA) versions. The results consist of the following plots: a) 2D plot, and b) Correlation Circle plot. In addition, the user can select the number of components for the computation, the number of variables to use in the sparse version, and the group variable.

1.1.4 Statistical Analysis

The Statistical Analysis task includes two modules: Differential Analysis and Enrichment Analysis.

The **Differential Analysis** module applies statistical algorithms to identify lipids with a different abundance among samples associated with experimental conditions (i.e., treatment versus control). The user can select between one of the two SE objects obtained from the previous steps (i.e., the one with the lipid abundance of all samples or where the technical replicates are averaged). The normalized corrected concentrations do not require further normalization when the experiment includes ISs. Instead, when the experiment does not incorporate ISs, it is possible to normalize the data matrix by a scaling factor at this stage. Then, the user can use linear models to compare each lipid abundance (in log scale) to several experimental factors. ADViSELipidomics uses the *limma* model for performing the statistical analysis and supports the user in defining the experimental design. Therefore, it allows fitting both simple and complex experimental designs. A complex design of ADViSELipidomics can include up to two experimental conditions and at most two variables to consider as batch effects. The user can choose the experimental variables from the columns of the Target File. Moreover, when a data matrix has technical replicates, the user can also incorporate the replicate effect in the model. More in detail, ADViSELipidomics copes with the batch effects by either fitting the model with the batch variables (i.e., user-selected or estimated as surrogate variables) or removing the batch effect before fitting the model. ADViSELipidomics uses the *removeBatchEffect* function from the limma package or the ComBat function (parametric or non-parametric method) from the SVA package to handle the batch effect. Note that the non-parametric *ComBat* approach can be very time-consuming. Before running the differential analysis, the user can define the contrasts list of interest (i.e., the conditions to compare) and select a threshold for the adjusted p-values. Then, ADViSELipidomics returns a table of results for each comparison, the MA-plot, and the Volcano plot. Moreover, ADViSELipidomics also performs pairwise comparisons between different contrasts using the Venn diagram and the Upset plot. Finally, it reports the list of common lipids in tabular form.

The **Enrichment Analysis** module interprets the results of a differential abundance analysis. It builds lipid sets from the chemical features of the lipids: i.e., lipid classes, total chain length (the sum of all carbon atoms in the tails), total unsaturation (the sum of all the double bonds in the tails). After defining a ranking for the differential abundant lipids (i.e., ranking considering logarithmic Fold Change, p-value, adjusted p-value, or B statistic), it identifies enriched sets of lipids using the permutation test in the *fgsea* function from the *fgsea* R package.

***1.2 Shiny App Interface***

ADViSELipidomics has a graphical user interface (GUI) implemented using *shiny* and *golem* R packages. Figure 3 shows the homepage with main sections in the menu bar: **Home**, **Data Import & Preprocessing**, **SumExp Visualization**, **Exploratory Analysis**, and **Statistical Analysis**. Moreover, the homepage includes a brief description of the software, the link to a GitHub repository for bug reporting, the reference to this publication for citation purposes, the link to the user’s manual, and the acknowledgment to project ADViSE. The **Home** section appears at the startup of ADViSELipidomics as homepage.


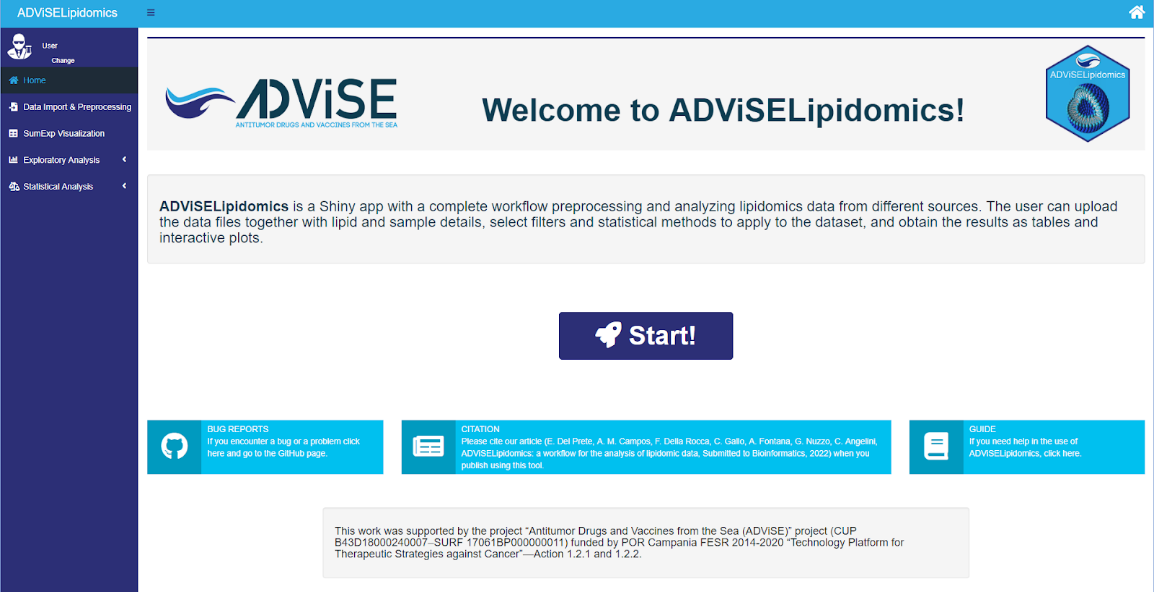


**Figure 3. Home section of ADViSELipidomics.** The Home section is the homepage of ADViSELipidomics. All the other main sections are listed in the menu bar on the left.

Figure 4 shows the **Data Import & Preprocessing** section, with the different parts enlightened with red rectangles.


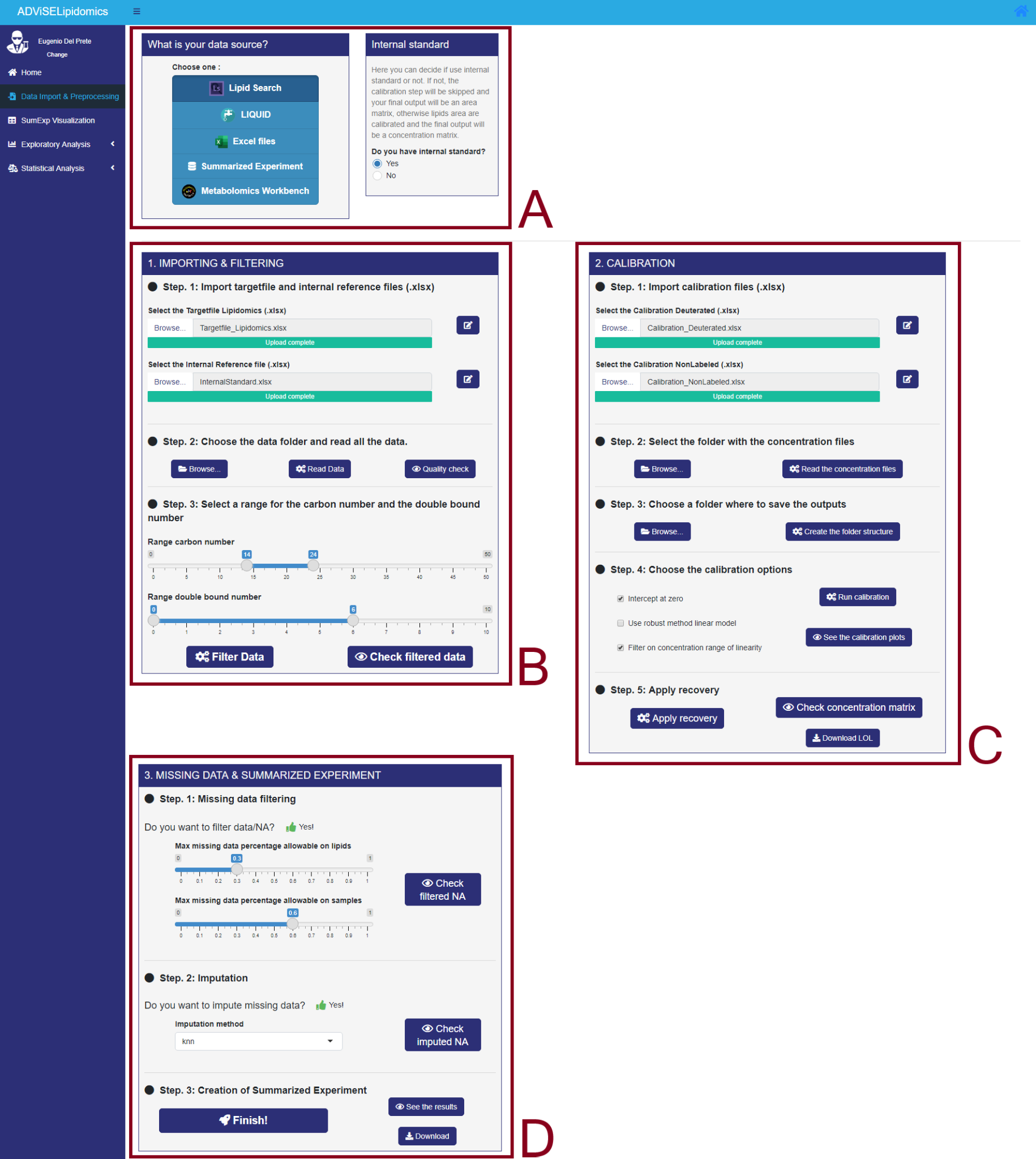


**Figure 4. Data Import and Preprocessing section of ADViSELipidomics.** Rectangle A provides the selection of different input data (with or without ISs). Rectangle B concerns the uploading of files, the storage folder, and the filtering parameters. Rectangle C is related to the calibration in the presence of ISs. Rectangle D copes with the missing data and the creation of the Summarized Experiment object.

Rectangle A allows the user to choose among different input formats: LipidSearch, LIQUID, Excel files, Summarized Experiment, and Metabolomics Workbench. In the case of LipidSearch data, it is also possible to select if ISs are present or not. Rectangle B shows three different steps for Importing & Filtering: importing data, storing and reading data, and filtering data. Rectangle C shows five additional steps for Calibration: importing calibration files, storing calibration files, selecting the folder for the results, selecting calibration options, and applying recovery. Finally, rectangle D shows two different steps for Filtering and Missing Data imputation and creating the SE object. Note that the layout of the Data Import & Preprocessing section depends on the type of input data format that the user chooses. Figure 4 illustrates the selection of LipidSearch data with ISs.

The **SumExp Visualization** section (figure not shown) allows the user to investigate every feature of the SE object, i.e., the lipid abundance data matrix, the annotation of lipids and samples, and metadata. The SE object is available in two formats: with all samples and with averaged technical replicates (if replicates are present).

The **Exploratory Analysis** section consists of three panels: Plots, Clustering, and Dimensionality Reduction. The Plots panel has the following subpanels, Lipids, Scatterplot, Heatmap, and Quality Plots, and each of them visualizes specific features. The panel Clustering provides hierarchical and partitioning clustering methods, with the possibility of tuning several parameters. Finally, the panel Dimensionality Reduction contains the subpanels PCA, PLS-DA, and sPLS-DA. Figure 5 shows PLS-DA, with X-Y variates plot on the left (rectangle A) and a correlation circle plot on the right (rectangle B). The user can select the variable of interest and the number of components and apply the PLS-DA to original or averaged data.


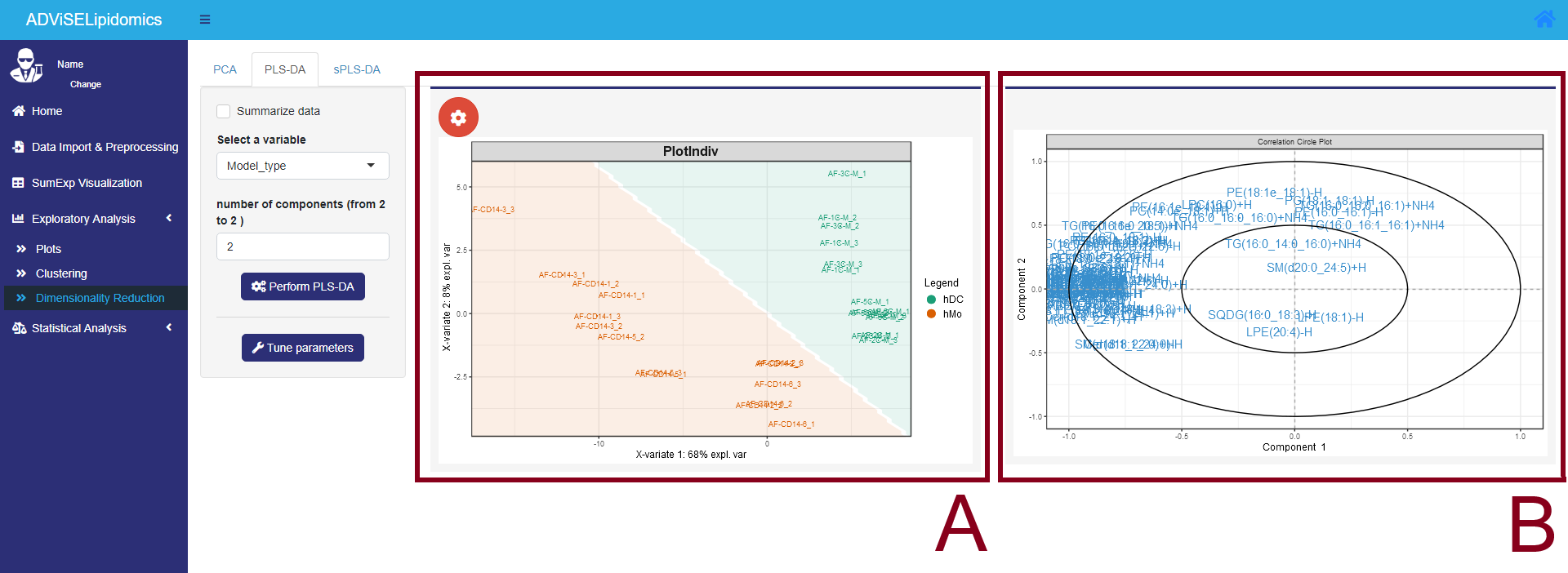


**Figure 5. Dimensionality Reduction panel from Exploratory Analysis section of ADViSELipidomics.** Rectangle A shows the PLS-DA plot, rectangle B shows the correlation circle plot for PLS-DA.

The **Statistical Analysis** section includes two panels: Differential Analysis and Enrichment Analysis. The panel Differential Analysis has the subpanels Build DA and Comparisons, with many options related to the definition of the experimental design, considering contrast features of interest, batch effects, and other parameters (e.g., adjusted p-value threshold). Figure 6 shows the Comparisons subpanel, with the Venn Diagram on the left (rectangle A) and the contrast list of the comparisons in tabular form on the right (rectangle B). The lipids reported in the table refer to the SwissLipids repository by using hyperlinks. The user can depict the same information with the Upset plot. The panel Enrichment Analysis takes the results of differential analysis in input and shows the statistical significance of the enrichment for different lipid sets based on lipid class, total chain length, and the total number of double bonds (examples are shown in Section 3).


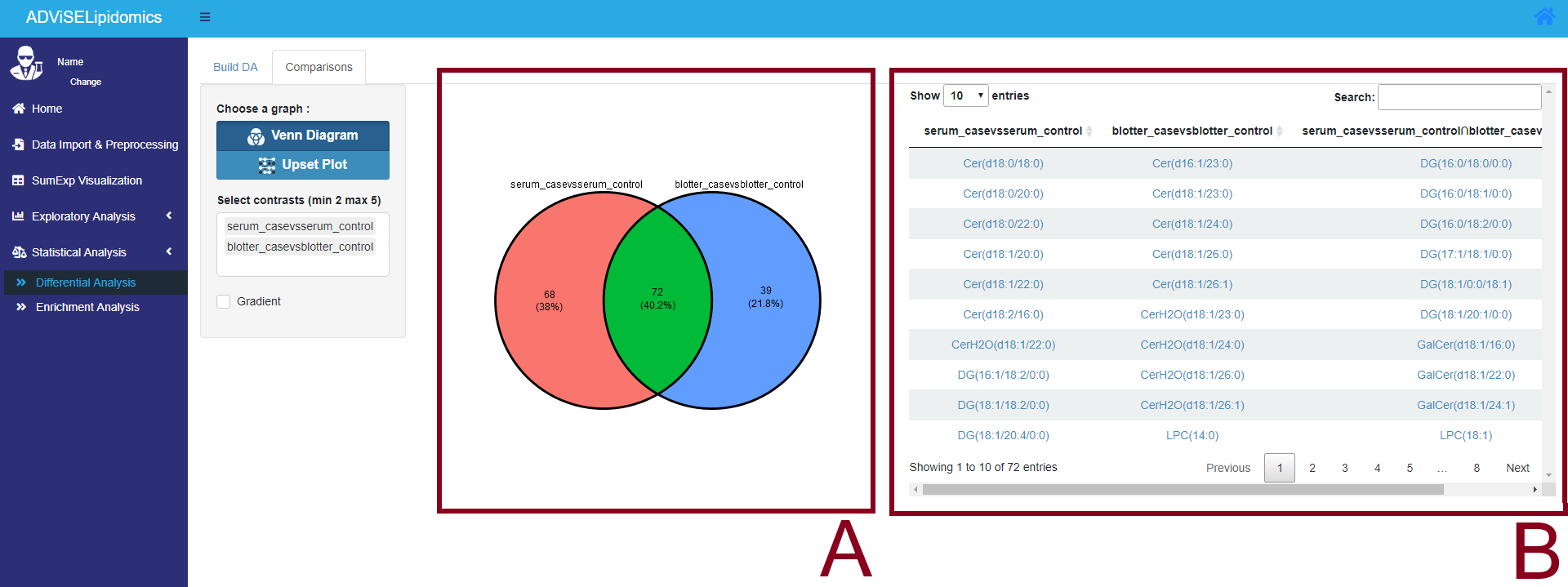


**Figure 6. Comparisons panel from Differential Analysis section of ADViSELipidomics.** Rectangle A depicts the Venn diagram among the contrasts on the left. Rectangle B shows the list of lipids per contrast, with the hyperlinks to SwissLipids.

***1.3 Type of Data***

1.3.1 Input

ADViSELipidomics allows importing several lipidomics files, depending on the software used to identify and quantify the lipids or already prepared datasets. Here we describe the possibilities:

- *LipidSearch with ISs*. In the Reading module, the user should upload a Target File (XLSX file) with mandatory variables, such as the list of the samples (or replicates per sample), information on the samples (e.g., condition, time, dose, product treatments, etc.), normalization factor, and optional variables, such as metadata (e.g., laboratory name, referent name, experiment date). At the same time, the user should upload an Internal Reference File (XLSX file) with the list of the lipids as ISs, defined per class and adduct, upper/lower bounds for the number of carbon atoms, upper/lower bounds for the number of double bonds, nominal standard concentration, and upper/lower bounds for the concentration linearity in the calibration curves. Then, the user selects the folder with the data files (TXT files) as obtained from LipidSearch, where a deuterated file and a non-labeled file represent each sample (or replicate). In the Calibration module, the user should upload a calibration deuterated file (XLSX file) and a calibration non-labeled file (XLSX file), both with the correspondence among concentration values, lipid classes, and concentration filenames. Finally, the user should select the folder with the concentration files (TXT files) created with LipidSearch. After importing, the lipid abundance is expressed as normalized corrected concentration value per lipid;
- *LipidSearch without ISs*. The files to upload in the Reading module are the same as in the previous case, whereas the Calibration module is absent and does not require any files. After importing, the lipid abundance is expressed as chromatographic peak area per lipid;
- *LIQUID*. In the Reading module, the user can upload a Target File (XLSX file) with the list of the samples (or replicates per sample) and related information, and an Internal Reference File (XLSX file) with the abovementioned upper/lower bounds defined per lipid class. Then, the user selects the folder with the data files (TSV files) obtained from LIQUID, where one file represents a sample (or replicate). After importing, the lipid abundance is expressed as peak intensity per lipid;
- *User’s Excel file*. In the Reading module, the user can upload a Target File (XLSX file) with the list of the samples (or replicates per sample) and related information, and a Data Matrix file (XLSX file) with data in a format suitable for the analysis: list of the lipids in the first column, necessary for the Parsing module, samples (or replicates) in the following columns. After importing, the lipid abundance is expressed as chromatographic peak area, peak intensity, or concentration value per lipid, depending on the scale of the Data Matrix file;
- *SummarizedExperiment*. The user should upload a SE object (RDS file) already prepared or previously downloaded after running ADViSELipidomics. We recommend being careful in the creation of the SE object, which consists of three main parts: rowData (lipids information), colData (samples or replicates information), and assay (data matrix). Data can be expressed as peak area, peak intensity, or concentration value per lipid;
- *Metabolomics Workbench*. The user selects a Metabolomics Workbench ID study. Then, ADViSELipidomics downloads the corresponding experiment and converts it into an SE object.

Further details on the structure of the files and a few downloadable examples are given in the user’s manual, present in the GitHub repository (see Section 1.5)

1.3.2 Output

ADViSELipidomics provides the results as tables and plots. After the uploading, the user can edit (if necessary) the Target File, the Internal Reference File, the Calibration Files, and download the new versions in XLSX format. Moreover, the user can visualize each part of the SE object and download the entire object as an RDS file, with the possibility to reload it into the app. ADViSELipidomics allows downloading all the plots in the Exploratory Analysis and Statistical Analysis steps as PNG files. Note that the plots are static once downloaded. When visualized in ADViSELipidomics, the graphs are interactive thanks to the *plotly* R package, allowing the user to move the cursor and select specific areas. A little window can show additional information on the selected region of interest in the interactive plots. Moreover, the table with differentially abundant lipids in the Venn Diagram holds hyperlinks to the SwissLipids online repository.

***1.4 R packages***

We implemented ADViSELipidomics using the R language by wrapping newly implemented R-functions with several existing R packages for the internal code and the user interface. ADViSELipidomics has a modular structure, where each module performs different tasks. This architecture allows the insertion of new functionality without overturning the workflow.

R packages dependencies are available in Bioconductor (Huber et al., 2015), Comprehensive R Archive Network, and GitHub repositories. Dependencies are automatically handled during installation. In the following, we report the main R packages dependencies in alphabetical order: *cluster* provides methods for cluster analysis (Maechler et al., 2021); *ComplexHeatmap* provides a flexible way to depicts multiple heatmaps and supports various annotations (Gu et al., 2016); *factoextra* facilitates the extraction and the visualization of the output from exploratory multivariate data analyses (Kassambara et al., 2020); *fgsea* implements an algorithm for fast gene set enrichment analysis, with more accurate p-values (Korotkevich et al., 2019); *golem* is a toolkit for simplifying the creation, development and deployment of a shiny application (Fay et al., 2021); *limma* allows data analysis, linear models, and differential expression for microarrays and other typologies of data (Ritchie et al., 2015); *lipidR* implements a workflow for downstream analysis of targeted and untargeted lipidomics data (Mohamed et al., 2020); *metabolomicWorkbenchR* provides functions for interfacing with the Metabolomics Workbench RESTful API (Lloyd et al., 2021); *mixOmics* provides unsupervised and supervised methods for the analysis and the integration of multivariate datasets (Rohart et al., 2017); *plotly* generates interactive web-based graphs via the open source JavaScript graphing library plotly.js (Sievert, 2020); *shiny* facilitates the building of interactive web application in R language (Sievert, 2020; Chang et al., 2021); *SummarizedExperiment* creates objects suitable for storing and analyzing biological experiment with information on data and samples (Morgan et al., 2021); *sva* provides algorithms for removing batch effects or other unwanted variation in high-throughput experiment, by creating surrogate variables (Leek et al., 2012); *tidyverse* is a collection of R packages designed explicitly for data science (Grolemund et al., 2017); *VIM* provides tools for the visualization of missing and/or imputed values with a view to the exploratory data analysis (Kowarik et al, 2016).

***1.5 Project Links***

ADViSELipidomics is freely available on GitHub at the following link: [*https://github.com/ShinyFabio/ADViSELipidomics*](https://github.com/ShinyFabio/ADViSELipidomics). The GitHub homepage has a brief description of the software, a guide that leads the user through the steps required for the installation, a user’s manual that helps in using ADViSELipidomics, and a short video on ADViSELipidomics functionalities.

More in detail, the user’s manual is available at the following link: [*https://shinyfabio.github.io/ADViSELipidomics_book/*](https://shinyfabio.github.io/ADViSELipidomics_book/). It describes the steps required for the installation of ADViSELipidomics, it guides the user through the different panels, and it provides the description for each file required by the software.

The example data for the Case Study One (see Sections 2.1, 4.2) can be downloaded from [*https://github.com/ShinyFabio/ADViSELipidomics_book/raw/main/data_example/Case_Study_%231.zip*](https://github.com/ShinyFabio/ADViSELipidomics_book/raw/main/data_example/Case_Study_%231.zip)

**2. Case Studies**

***2.1 Case Study One***

Lipids are biologically active and ubiquitous metabolites displaying different functions within the cells (Wolrab et al., 2020; Satomi et al., 2017; Zhao et al., 2015). The development of pathologies or the physiological state of a cell is often observed in the lipid profile of plasma and/or cells, and the accurate assessment of lipid profiles can allow for the discovery of lipid biomarkers (Coillard et al., 2019). As previously reported, circulating monocytes can infiltrate mucosal or inflamed tissues and differentiate into dendritic cells (Coillard et al., 2019; Tang-Huau et al., 2018). The study of the adaptation of the lipid profile of the cells during the induced differentiation from monocytes to immature dendritic cells (iDCs) allows us to better understand the natural process occurring in human tissues.

The dataset consisted of 30 samples divided into two study groups: primary human monocytes (hMos) and iDCs. Each study group has 5 samples with 3 replicates. We generated the mass spectra on a Q-Exactive Hybrid Quadrupole – Orbitrap mass spectrometer (Thermo Scientific, San Jose, CA, USA), equipped with an ESI source coupled to an Infinity 1290 UHPLC System (Agilent Technologies, Santa Clara, CA, USA). First, we performed lipid identification and relative quantification using LipidSearch 4.2.29 software (Thermo Scientific). Then, we used the LipidSearch as input files for ADViSELipidomics.

***2.2 Case Study Two***

Longitudinal studies are a helpful tool to diagnose the development of pathologies. Biofluids, usually serum and plasma extracted from blood, are collected during an extensive interval of time in order to understand the onset and development of disease-induced alterations. In an original study, researchers evaluated if dried blood spots (DBS) samples could be used in a longitudinal study following metabolic disease. They compared lipids identified in DBS samples to lipids of matched serum samples from a case-control study of older diabetic males.

The dataset is available in the Metabolomics Workbench online repository with the Study ID ST000608 and consists of 60 mass spectra divided into four different groups of the same size: 5 samples of serum from male control participants and 5 serum samples from diabetic males, in addition with 5 DBS matched control samples and 5 DBS matched samples from diabetic males. Each sample was run three times in LC-MS/MS. We focused on the lipid dataset from case and control serum samples to test the potential of ADViSELipidomics on public datasets.

**3. Results**

This section aims to demonstrate the capabilities of ADViSELipidomics in analyzing different case studies where data are obtained from different sources. Moreover, with the comparison between Case Study one with and without the usage of ISs, we also demonstrate the advantages of using ISs (as described in Section 3.1 and with analytical detail given in Section 4) for better quantification of lipid abundance.

***3.1 Improved LC-MS/MS Method***

In recent years, lipids gathered the attention of researchers. Lipids play essential biological roles from structural cell components to energy storage and signaling (Wolrab et al., 2020; Satomi et al., 2017; Zhao et al., 2015), both in physiological and pathological conditions. Often the development of pathologies is a consequence of the dysregulation of the lipid profile in plasma and/or other cells, as in cancer (Pakiet et al., 2021; Patterson et al., 2011), nonalcoholic fatty liver disease (Tiwari-Heckler et al., 2018; Puri et al., 2009), or neurological diseases (Luczaj et al., 2016; Luczaj et al., 2018). Therefore, the accurate assessment of lipid content in samples can identify subsets of lipids as biomarkers, or it can allow early-onset disease diagnosis by correlating the metabolism of lipids with the stage of the disease (Zhao et al., 2015; Luczaj et al., 2016; Luczaj et al., 2018; Colombo et al., 2018).

We selected Mass Spectrometry (MS) - based techniques to perform quantitative lipid analysis (Zhao et al., 2015). Here, we present an improved UHPLC- HRESIMS-MS/MS method for the absolute quantification of each lipid species identified from a wide range of lipid classes. We started from a method previously developed (Cutignano et al., 2016), as a benchmark for optimizing the UPHLC-HRESI method employed in this study. In this new experimental protocol, we used methanol and 0.005% ammonium hydroxide as an eluent system. Lipids were separated over a 40 min run by a gradient from 60% to 100% methanol in 15 min, followed by isocratic conditions for 15 min and then returned to initial running conditions (see Section 4). This optimization allowed a better distribution of different lipid classes and ionization of lipid species.

We selected 17 ISs (see Section 4.1) and validated the analytical method for each standard. The IS used are either deuterated or contain fatty acyl chains of seventeen carbons, and each standard represents a different lipid class. The selected IS are supposed to have similar extraction recoveries, ionization responses in electrospray ion sources, and approximate chromatographic RT as naturally occurring species of the corresponding lipid class, with the advantage of displaying an *m/z* value different from natural occurring lipid species of the same class. We evaluated selectivity, carry-over effect, the response of the instrument with the concentration of the IS, and established the corresponding lower limit of quantification. We also evaluated matrix effect, precision, and accuracy using quality control (QC) samples, with and without IS, and running intra and inter-day analysis. Overall, the improved analytical method showed great sensibility, and linearity for all the considered IS. The presence of matrix did not interfere significantly with the ionization of the different IS used, with a coefficient of variation (CV) associated with MF always less than 15%. Also, the analytical method displayed intra-run and between-run accuracy and precision with percentage error and CV less than 15% for most of the standards. The broad range of lipid classes capable of being accurately and reliably quantified by our improved LC-MS/MS method allows different typologies of natural matrices, from human cells to natural extracts, to be analyzed using a single LC-MS/MS method (data not shown).

***3.2 Case Study One***

3.2.1 Data Analysis using ISs

To study how the lipid profile adapts during induced differentiation from monocytes into iDCs, we analyzed the lipids of 5 samples of primary hMos and 5 samples of iDCs. The samples were collected in triplicate, for a total of 30 LC-MS/MS spectra. Before lipid extraction, we spiked all samples with a known amount of IS Mix. The lipids present on the IS Mix are detailed in Table 4 (see Section 4.1.1).

We used the same IS Mix to prepare a set of 11 calibration curve points in the range 0.1 ng/mL to 5 mg/mL by a series of dilutions. Each sample and calibration curve point were run in triplicate by LC-MS/MS on a Q-Exactive Hybrid Quadrupole – Orbitrap mass spectrometer equipped with an ESI source and coupled to an Infinity 1290 UHPLC System. We loaded the obtained LC-MS/MS spectra into LipidSearch 4.2.29, which identified lipids through the general and labeled databases. The lipid identification considered as adduct [M+H]^+^, [M+Na]^+^, [M+NH_4_]^+^ in positive mode, and [M-H]^-^ in negative mode, searching the following lipid classes: Cer, LPC, LPE, PC, PE, PG, PS, SM, DG, ChE, and TG. We uploaded the resulting text files into ADViSELipidomics, filtering by RT, number of carbon atoms, and number of double bonds accordingly. Finally, we built the calibration curves setting the intercept at zero and filtered according to the linearity range pre-established in the Internal Reference File. We obtained the normalized corrected concentration matrix. Then, we filtered the normalized corrected concentration matrix by removing lipids not present in at least 60% of samples and samples with more than 40% of missing/undetected lipids. The final SE object consisted of 86 lipid species for 30 mass spectra. Finally, we imputed the remaining NA values using the KNN method.

In Figure 7, from the 86 identified lipid species, the glycerophospholipids represent slightly over 70% of the identified lipids, being PC and PE the classes with the higher number of identified species.


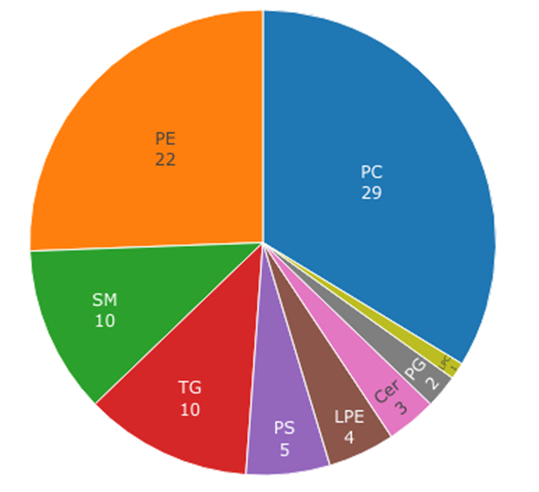


**Figure 7. Pie chart with the number of lipid species identified for each lipid class (Case Study One with ISs).** Each color identifies a lipid class. The highest abundances are for PC (29) and PE (22) lipid classes.

The PCA shown in Figure 8 differentiates primary hMos from iDCs. The two study groups are displayed separately.


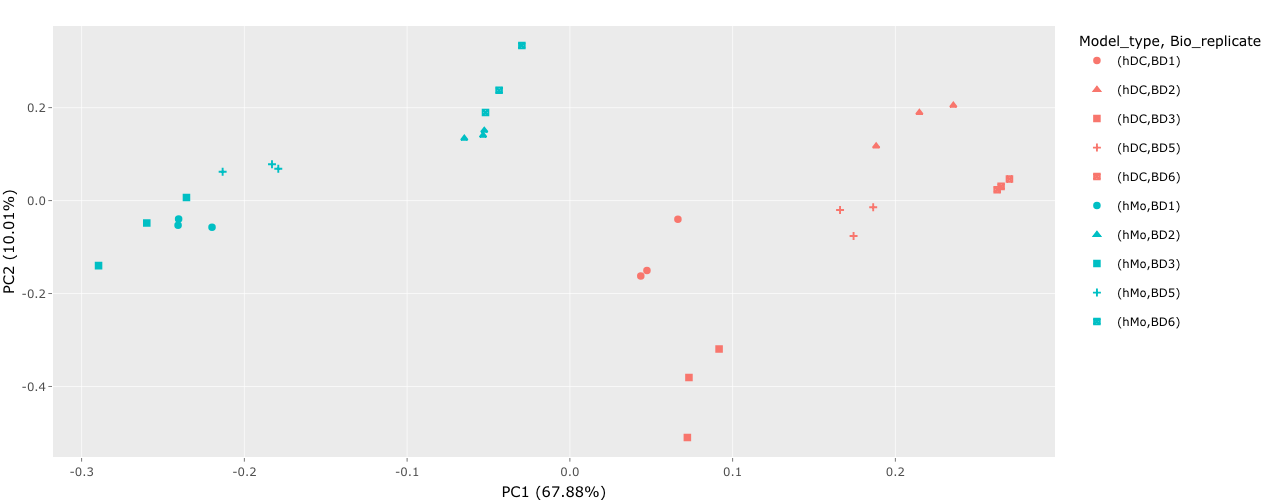


**Figure 8. PCA (Case Study One with ISs).** PCA plot defines samples by color and biological replicates by shape. hDCs and hMos are clearly separated, and all the biological replicates (BDx) are grouped in subsets of three.

The first principal component (PC1) represents 67.88% of the observed variability, indicating that the lipidome of monocytes and iDCs are considerably different. These differences can be further inferred by visualizing the proportion of each lipid class in each sample, as shown in Figure 9.


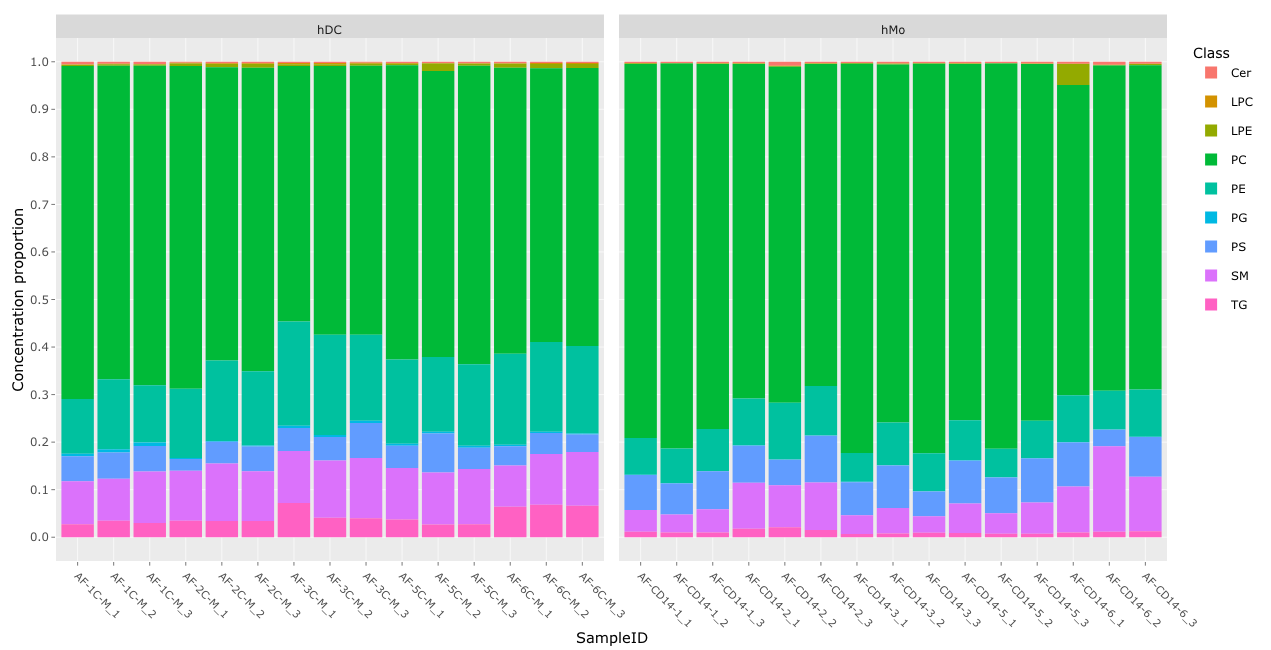


**Figure 9. Lipid class distribution in primary hMos and iDCs (Case Study One with ISs).** The two panels report the concentration in terms of proportion for both the conditions, with stacked bars colored per class. The AF-CD14-6-1 sample has the highest abundance of LPE, compared to the other samples.

PC represents approximately 70 – 80% of the lipidome in hMo, whereas there is an abundance reduction of approximately 10% in iDCs, along with an abundance increase in PE, SM and TG. The heatmap in Figure 10 illustrates the same trend.


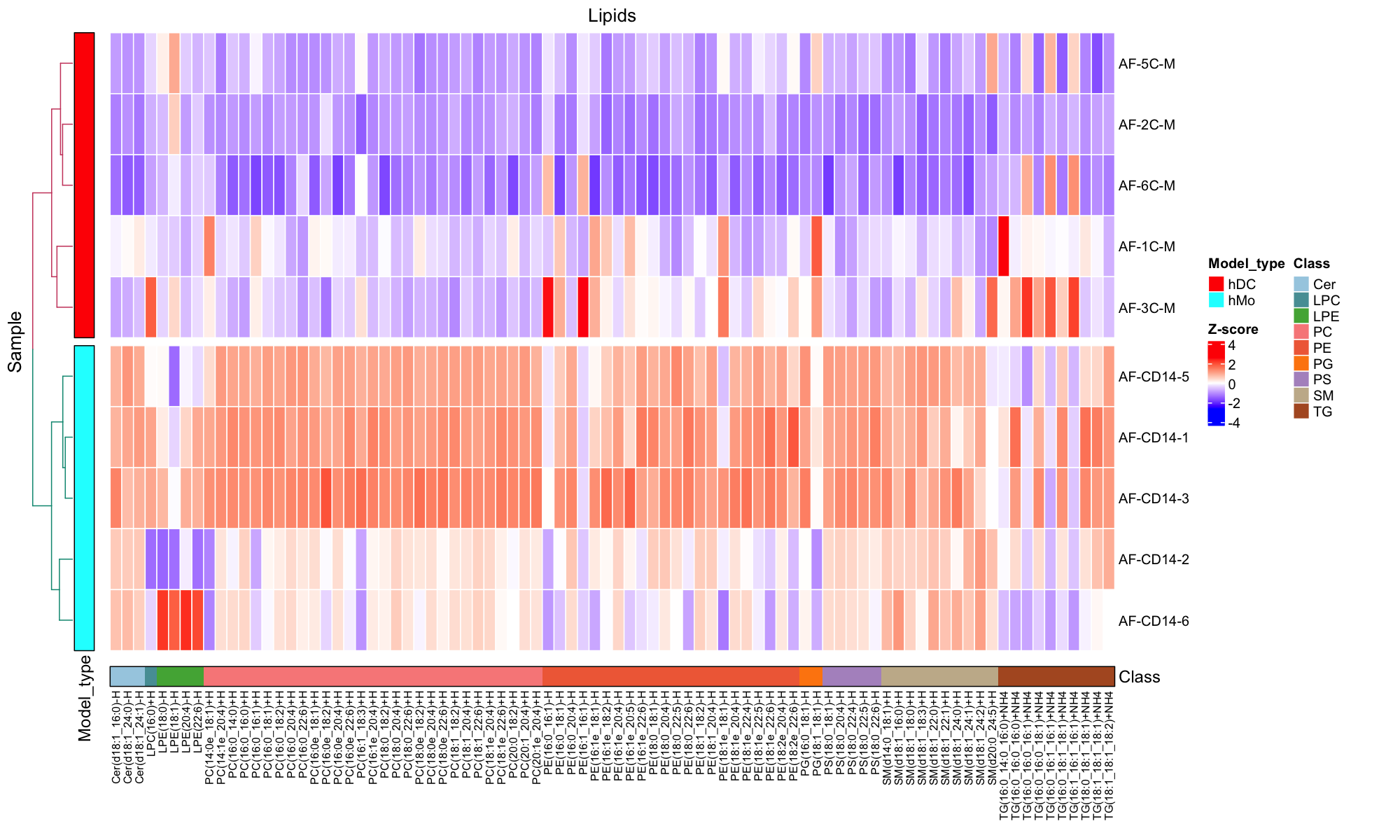


**Figure 10. Heatmap with the abundance of each identified lipid species (Case Study One with ISs).** The figure shows a perfect clustering between hDC (red) and hMo (light blue). Abundance values are converted to Z-score, with a gradient from high negative values (dark blue) to high positive values (dark red). Lipid classes are ordered per color: we can underline a small different behavior for LPE (green) and some TG (brown).

According to the abundance of each lipid species, iDCs samples AF-1C-M and AF-3C-M display a lipidome profile slightly different from the remaining iDCs samples. Primary hMo samples AF-CD14-2 and AF-CD14-6 have similar behavior, displaying a slightly different lipidome than the remaining primary hMo samples. These differences between samples could explain the variability within each condition observed with the PCA. Nonetheless, as previously observed for the distribution of each lipid class per sample, the heatmap displays an increased amount of PC, PE, SM, and PS species in primary hMos compared to iDCs. TG species show a higher variation. We also performed differential analysis considering Summarized data, without normalizing replicates or samples, selecting as primary variable Model_type, considering batch effect (Batch type - remove; Batch method - limma; Primary batch variable - Bio_replicate*),* p-value threshold of 0.05 and separate method for *decideTests* R function. The data are represented as a volcano plot in Figure 11 and as a list of differential abundant lipids in Table 1, and they confirm the differences already observed.


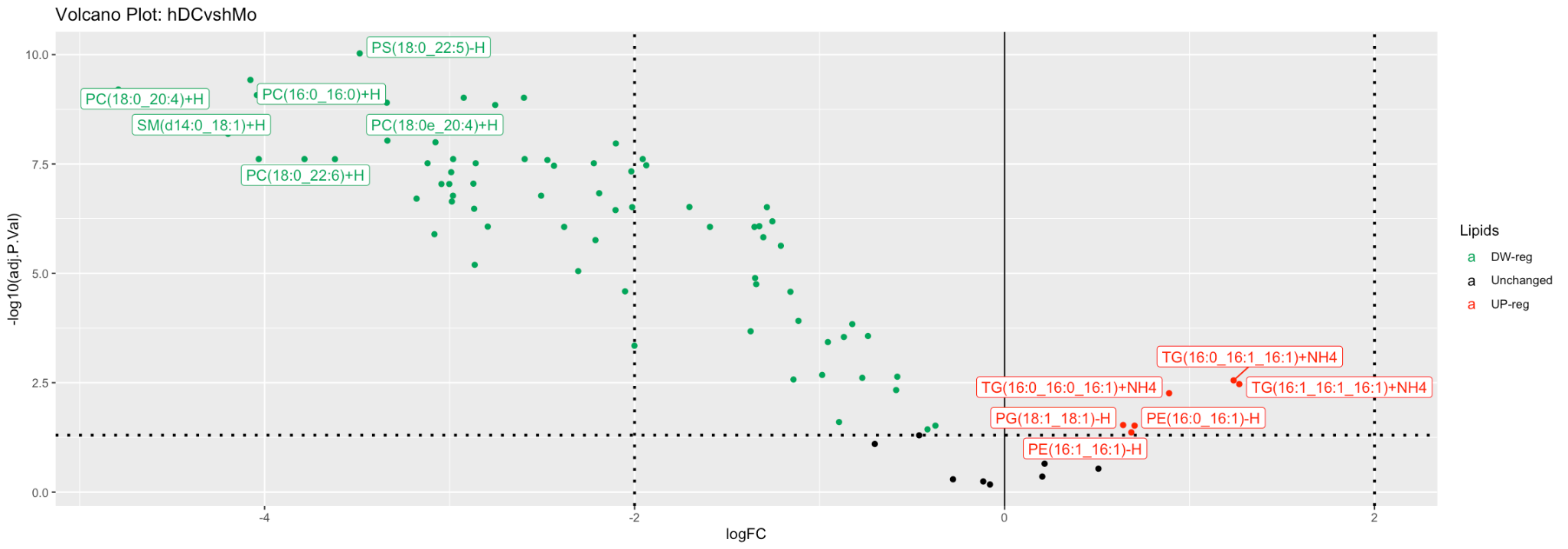


**Figure 11. Volcano plot with differential analysis of lipid species between primary hMo and iDCs (Case Study One with ISs).** The volcano plot depicts down-regulated lipid species (green) and up-regulated lipid species (red).

More in detail, 78 lipid species are statistically different between monocytes and iDCs, where most of the species are less abundant in the lipidome of iDCs. Only 6 lipid species belonging to PE, PG and TG are more abundant in iDCs than in monocytes.

**Table 1. List of differential analyzed lipid species between hDC and hMo samples (Case Study One with ISs).** The values in the table represent the 78 differential analyzed lipid species shown in Figure 11. The first column is the logarithmic fold change (logFC), the second column is the adjusted p-value (adj.P.value), the third column is the sign of the regulation (DE), the fourth column is the lipid class (Class), and the fifth column is the lipid species (Lipids). The list is in alphabetical order by lipid class, and the column DE defines down-regulated lipid species (-1) and up-regulated lipid species (1).


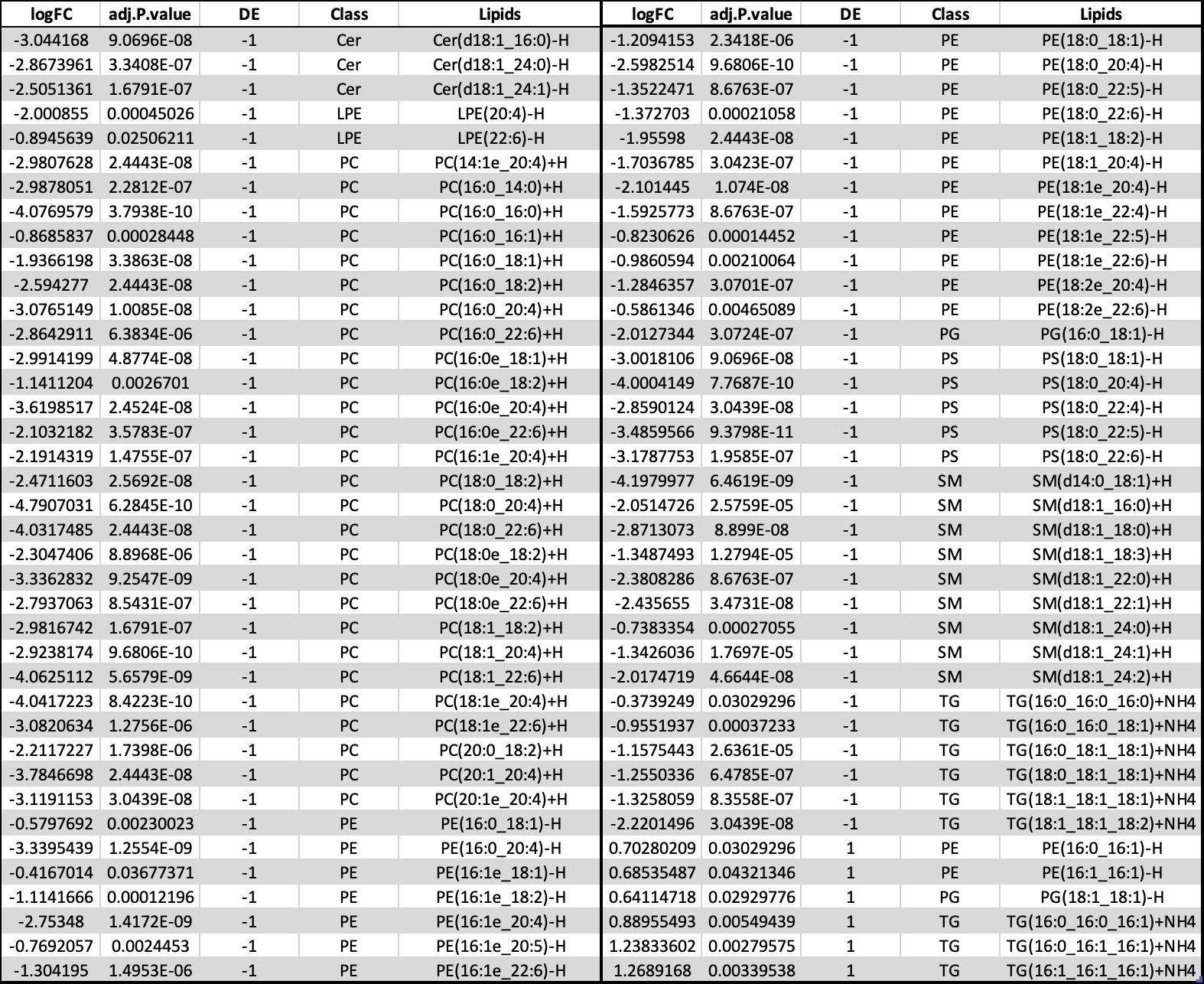


Figure 12 depicts the enrichment analysis, reinforcing the evidence that PC species are significantly less abundant in iDCs. Moreover, we observed an overall higher amount of lipid species with a total number of 48 carbons in iDCs, which agrees with the identified TG species, more expressed in iDCs. Unsaturation does not provide statistically significant differences.


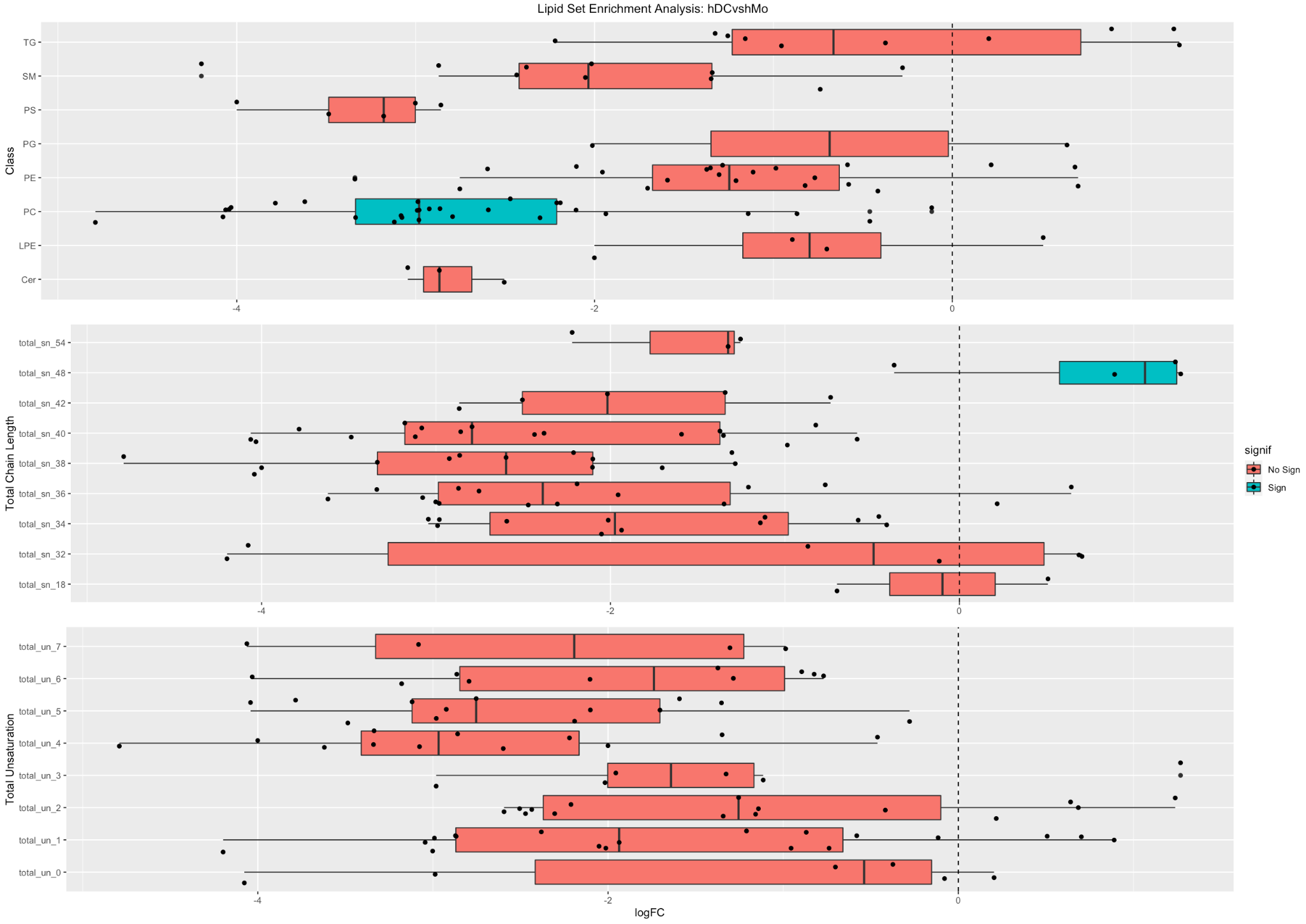


**Figure 12. Lipid Set Enrichment Analysis (Case Study One with ISs).** The first panel shows the boxplots separated for lipid class, the second panel shows the boxplots separated for total chain length, and the third panel shows the boxplots separated for total number of unsaturated bonds. PC is significantly enriched in the first panel, total chain length of 48 is significantly enriched in the second panel (light blue). The number of double bonds does not provide statistically significant evidence. Legend: No Sign, absence of statistical significance; Sign, presence of statistical significance; total_sn_xx, total chain length; total_un_yy, total number of double bonds (unsaturation).

The number of studies focused on the lipidomic profile of DCs is considerably low. A study reported that maturated fetal skin-derived DCs displayed increased levels of Cer and PC, along with reduced content of PI and SM (Santinha et al., 2012). In a more in-depth study, researchers studied the lipidomic profile changes of murine bone-marrow-derived tolerogenic versus immunogenic DCs (Ocaña-Morger et al., 2017). Immunogenic DCs displayed increased Cer levels, while tolerogenic DCs were characterized by increased levels of ChE and plasmalogen PC. A more recent study analyzed the lipidome of hMo-derived DCs during maturation, i.e., from immature to mature dendritic cells (Luhr et al., 2020). Maturation of DCs led to a significant overall change in the lipid profile of the cells characterized by an increase in storage lipids and differences in the relative abundance of cellular membrane lipids. Overall data indicate that the profile of dendritic cells is highly susceptible to change. However, no study has ever focused on the lipidomic changes during induced differentiation from monocytes into iDCs. A study reported that monocyte-induced differentiation into DCs was accompanied by up-regulation expression of genes related to lipids' metabolism (Le Naour et al., 2001), which suggests that changes also occur during differentiation at the lipidome level. To our knowledge, the study presented in this manuscript is the first that unravels those changes. Interestingly, as already mentioned, among the lipid species less expressed in iDCs, the ones with a more pronounced logFC correspond mainly to PC species with long and polyunsaturated fatty acyl chains. By contrast, the more expressed lipid species display shorter and saturated or monounsaturated fatty acyl chains (Table 1), suggesting that the cell membrane of monocytes is more fluid than the cell membrane of dendritic cells.

3.2.2 Data Analysis without using ISs

This section re-analyzes the Case Study One data, assuming that the experiment was without ISs. The aim is twofold. From one side, we show how ADViSELipidomics can handle experimental data without ISs. On the other hand, we show the advantages of using ISs, leading to a better quantification of lipid abundances.

We submitted the previous dataset of 30 LC-MS/MS spectra to LipidSearch 4.2.29 for lipid identification against the internal database, following the same search criteria reported in Section 3.2.1. Then, we directly uploaded the resulting TXT files into ADViSELipidomics and filtered them by RT, number of carbon atoms, and number of double bounds. In this case, ADViSELipidomics used chromatographic peak area per lipid as lipid abundances (without performing the calibration and recovery steps).

As in Section 3.2.1, we filtered out lipids and samples using the same criterion before applying the missing data imputation with the KNN method. The final SE object consisted of 86 lipid species and 30 mass spectra.

As expected, without the presence of IS, the lipid quantification is less accurate. In fact, glycerophospholipids represent over 70% of the total number of identified lipid species, with PC and PE the most abundant species (data not shown). As observed for the normalized data, the non-normalized data shows a clear spatial separation between monocytes and iDCs when plotting the data as a PCA plot (data not shown). Moreover, the proportion of each lipid class in the lipidome of each sample is considerably different from the trend observed for normalized data. In this case, PC represents only 30 – 50% of the total lipidome of monocytes, while for iDCs PC ranges approximately from 60 to 70% of the total lipidome (Figure 13), in contrast to previously reported.


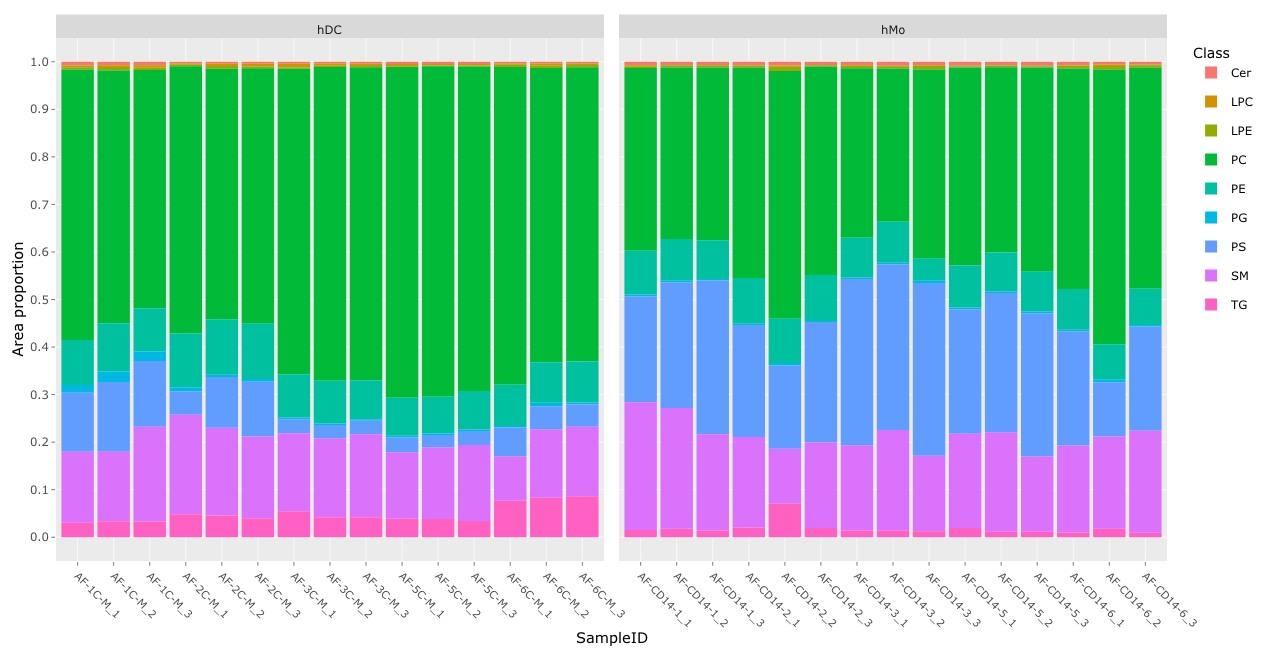

**Figure 13. Lipid class distribution in primary hMos and iDCs (Case Study One without ISs).** The two panels report the concentration in terms of proportion for both the conditions, with stacked bars colored per class. hDC cells display a higher abundance of PC than hMo cells.

This leads us to conclude that the absence of the calibration and recovery steps for normalized data with IS provides a wrong estimation of the abundance of each class in the samples. The considerably lower abundance of PC in monocytes seems to be due to a considerably higher estimated abundance of PS (which represents up to approximately 40% of the lipidome of monocytes). By contrast, in Section 3.1, it corresponds to 10%.

Nevertheless, in agreement with the discussed data above, the heatmap shows that most individual lipid species are more abundant in monocytes than iDCs, including PC species (Figure 14), agreeing with the previously observed data. Even without IS, monocyte samples AF-CD14-2 and AF-CD14-6 display a lipid profile slightly different from the remaining monocyte samples, as previously reported in Figure 10.


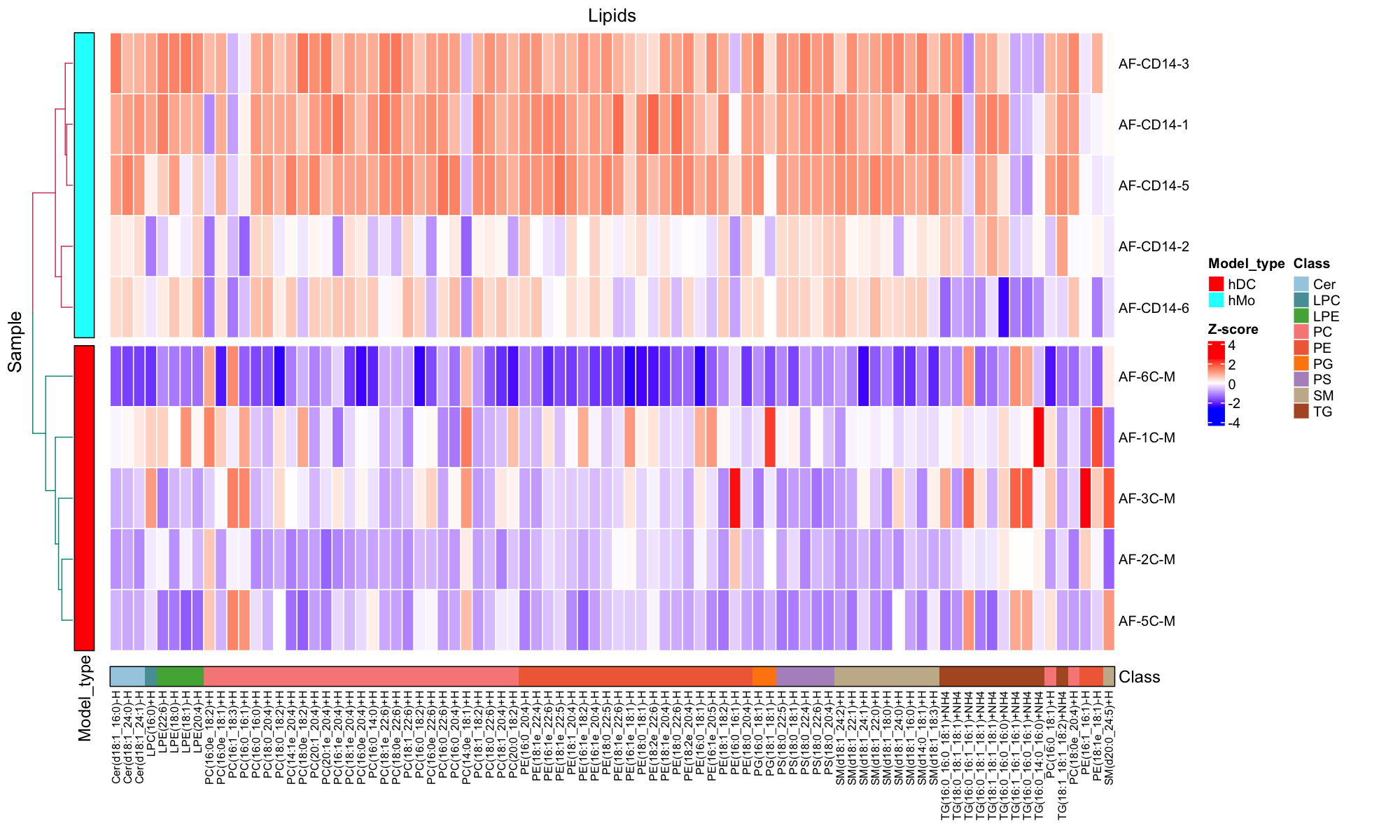


**Figure 14. Heatmap with the abundance of each identified lipid species (Case Study One without ISs).** The figure shows a perfect clustering between hDC (red) and hMo (light blue). Abundance values are converted to Z-score, with a gradient from high negative values (dark blue) to high positive values (dark red). Lipid classes are ordered per color: we can underline a small different behavior for some TG (brown) and PE (orange) species.

After running the differential analysis, a total of 57 lipid species display a statistically significant abundance between iDCs and monocytes. In the case of normalized data, there is a clear distinction between the lipid classes that are statistically significant, as detailed in the previous section. However, when the analysis is done without considering IS, different lipid species within the same class are responsible for the difference observed between monocytes and iDCs, as observed for some lipid species within PC and PE classes (Figure 15 and Table 2). Also, some PS species are significantly less abundant in iDCs than monocytes, which validates the differences in lipid class distribution observed on Figure 13.


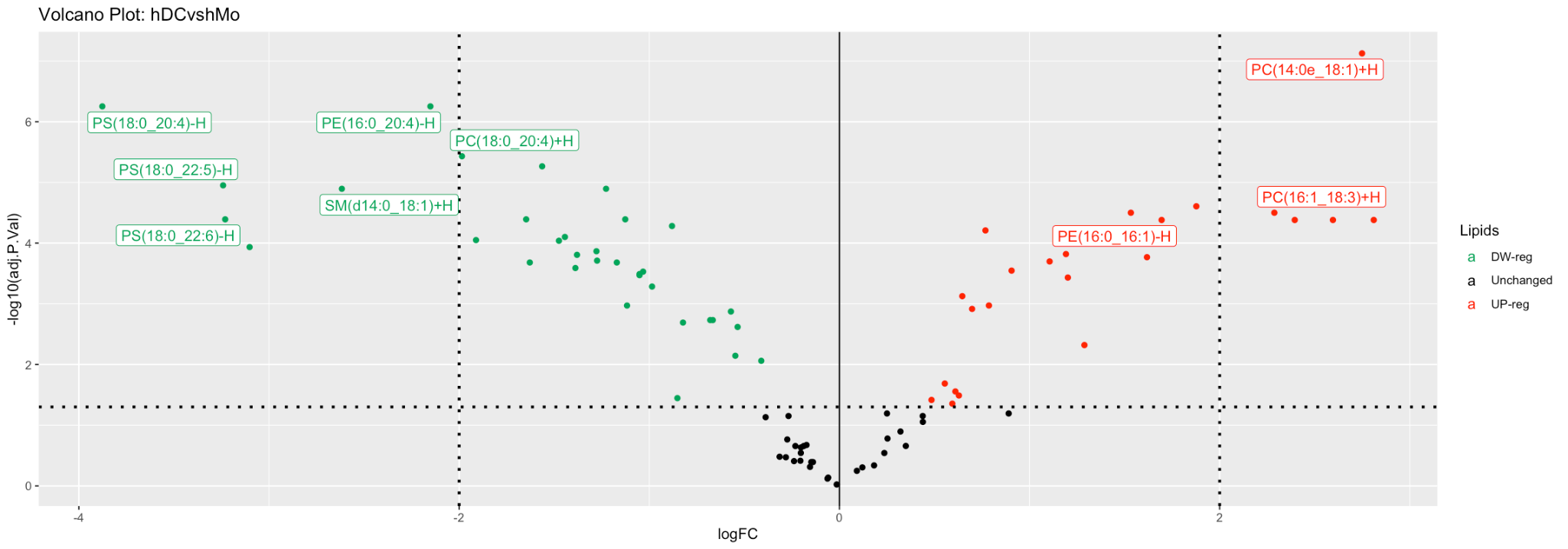


**Figure 15. Volcano plot with differential analysis of lipid species between primary hMos and iDCs (Case Study One without ISs).** The volcano plot depicts down-regulated lipid species (green) and up-regulated lipid species (red).

**Table 2. List of differential analyzed lipid species between hDC and hMo samples (Case Study One without ISs).** The values in the table represent the 57 differential analyzed lipid species shown in Figure 15. The first column is the logarithmic fold change (logFC), the second column is the adjusted p-value (adj.P.value), the third column is the sign of the regulation (DE), the fourth column is the lipid class (Class), and the fifth column is the lipid species (Lipids). The list is in alphabetical order by lipid class, and the column DE defines down-regulated lipid species (-1) and up-regulated lipid species (1).


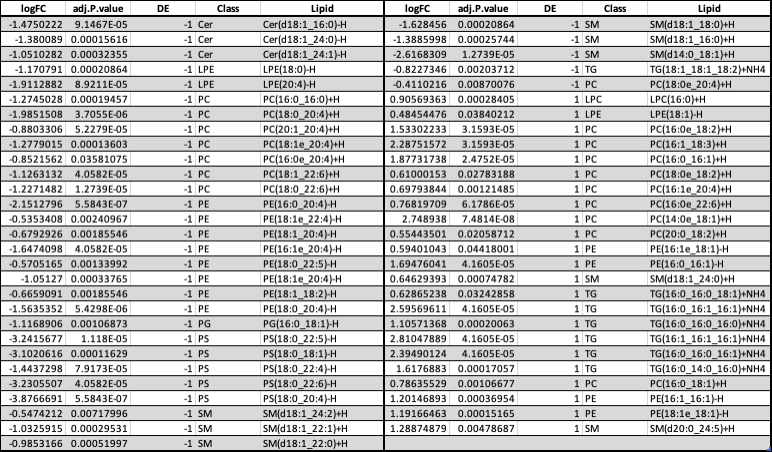


The enrichment analysis shown in Figure 16 confirms this difference, and does not highlight any other significant variations between monocytes and iDCs. From comparing results with the presence and the absence of IS for the same dataset, we found that it is still possible to distinguish between experimental conditions using non-normalized data. However, the identified lipid species responsible for the differences between conditions are less precisely quantified, justifying the necessity of using proper IS in MS lipidomic experiments.


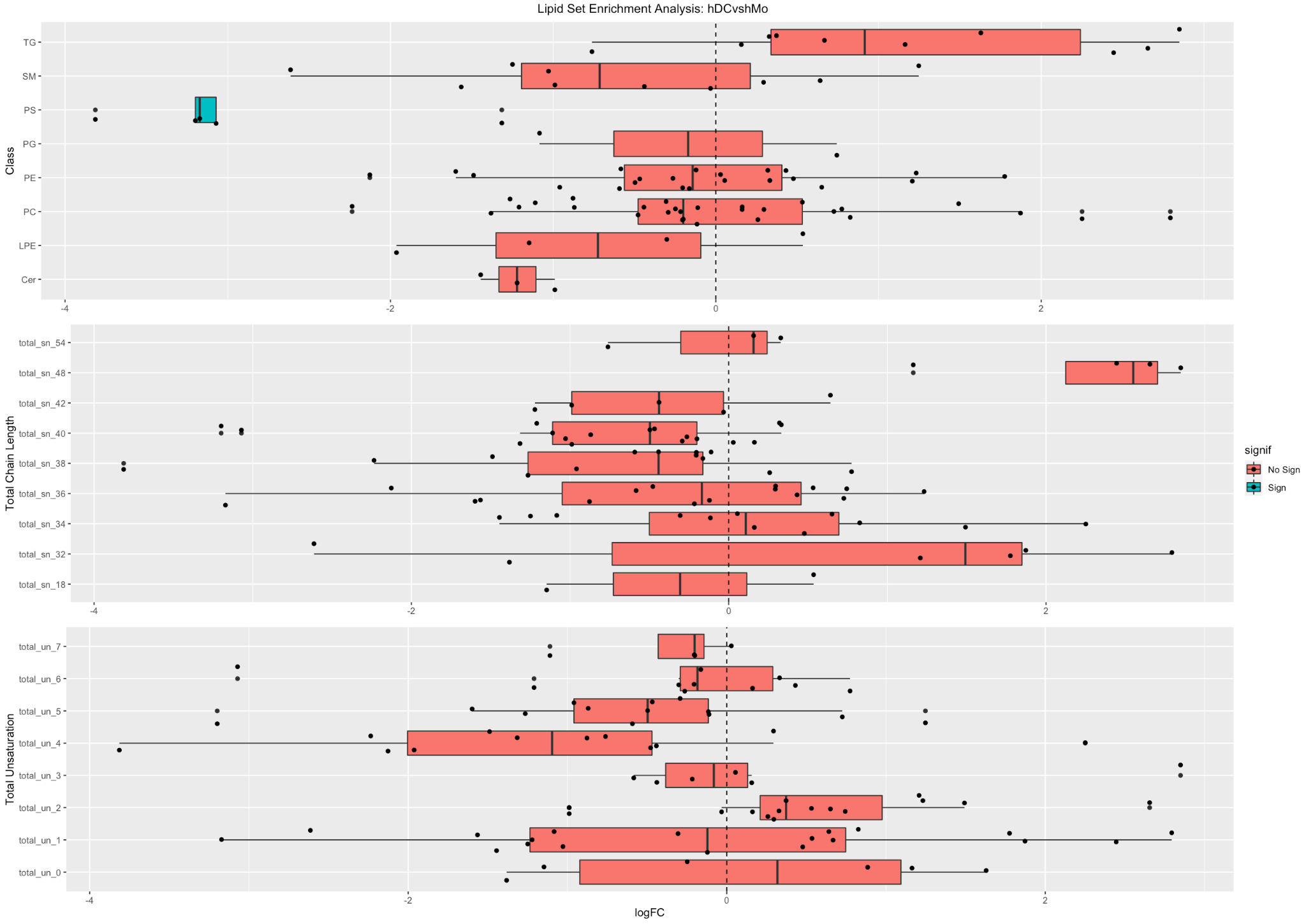


**Figure 16. Lipid Set Enrichment Analysis (Case Study One without ISs).** The first panel shows the boxplots separated for lipid class, the second panel shows the boxplots separated for total chain length, and the third panel shows the boxplots separated for total number of unsaturated bonds. PS is significantly less enriched in the first panel. The total chain length and number of double bonds does not provide statistically significant evidence. Legend: No Sign, absence of statistical significance; Sign, presence of statistical significance; total_sn_xx, total chain length; total_un_yy, total number of double bonds (unsaturation).

***3.3 Case Study Two***

The dataset available in Metabolomics Workbench (Study ID ST000608) consists of 60 mass spectra derived from comparing four different experimental conditions. There are 5 LC-MS/MS samples for each condition, with 3 technical replicates. Experimental conditions are: serum from control male participants, serum from diabetic male participants, DBS matched control samples, and DBS matched diabetic male samples. ADViSELipidomics provides the possibility of downloading the complete lipidomic dataset directly from the online repository. When filtering lipids and samples for missing values, we removed lipids not present in at least 60% of the samples and samples with more than 50% of missing/undetected lipids.

Then, we applied the imputation of the remaining missing values with the median method. We had a dataset of 274 lipid species for 60 samples, converted into a SE object. Here, for illustrative purposes, we compared case and control serum samples from the clean dataset to test the potential of ADViSELipidomics.

As reported in a study (Kyle et al., 2017), glycerophospholipids represent around 50% of the total number of lipid species identified in the serum, although they identify approximately the same number of TG and PC species, as shown in Figure 17.


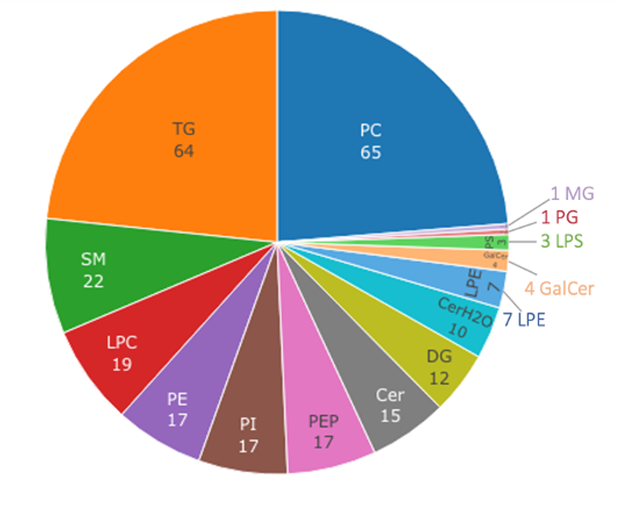


**Figure 17. Pie chart with the number of lipid species identified for each lipid class (Case Study Two).** Each color identifies a lipid class. The highest abundances are for PC (65) and TG (64) lipid classes.

Figure 18 and Table 3 show 163 lipids with a statistically significant differential abundance. Most of these lipid species are less expressed in case samples, especially glycerophospholipids, except for PE. On the contrary, most galactolipids are over-expressed in case samples, specifically DG and TG, according to the original study. Despite the different number of statistically significant lipid species, 163 identified by ADViSELipidomics versus 118 reported in the study, the species that were differently produced in serum case samples versus serum control samples belonged to the same lipid classes.


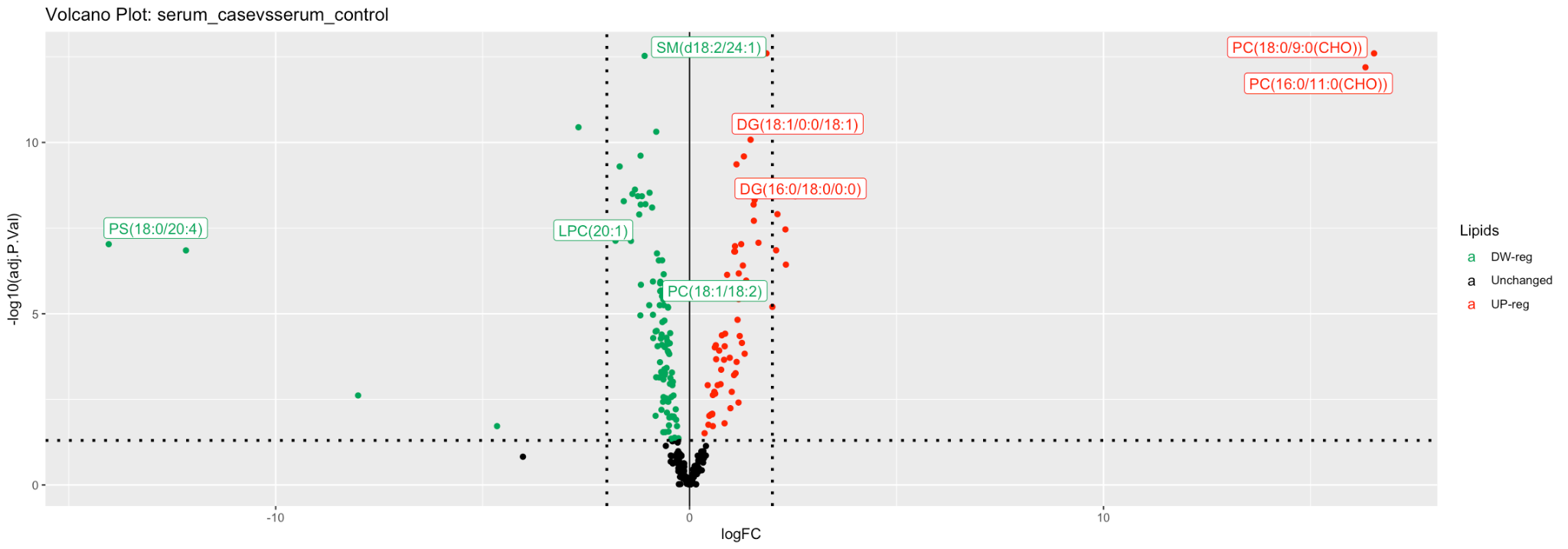


**Figure 18. Volcano plot with differential analysis of lipids species between case serum samples and serum control samples (Case Study Two).** The volcano plot depicts down-regulated lipid species (green) and up-regulated lipid species (red).

**Table 3. List of differential analyzed lipid species between control and diabetic serum samples (Case Study Two).** The values in the table represent the 163 differential analyzed lipid species shown in Figure 18. The first column is the logarithmic fold change (logFC), the second column is the adjusted p-value (adj.P.value), the third column is the sign of the regulation (DE), the fourth column is the lipid class (Class), and the fifth column is the lipid species (Lipids). The list is in alphabetical order by lipid class, and the column DE defines down-regulated lipid species (-1) and up-regulated lipid species (1).


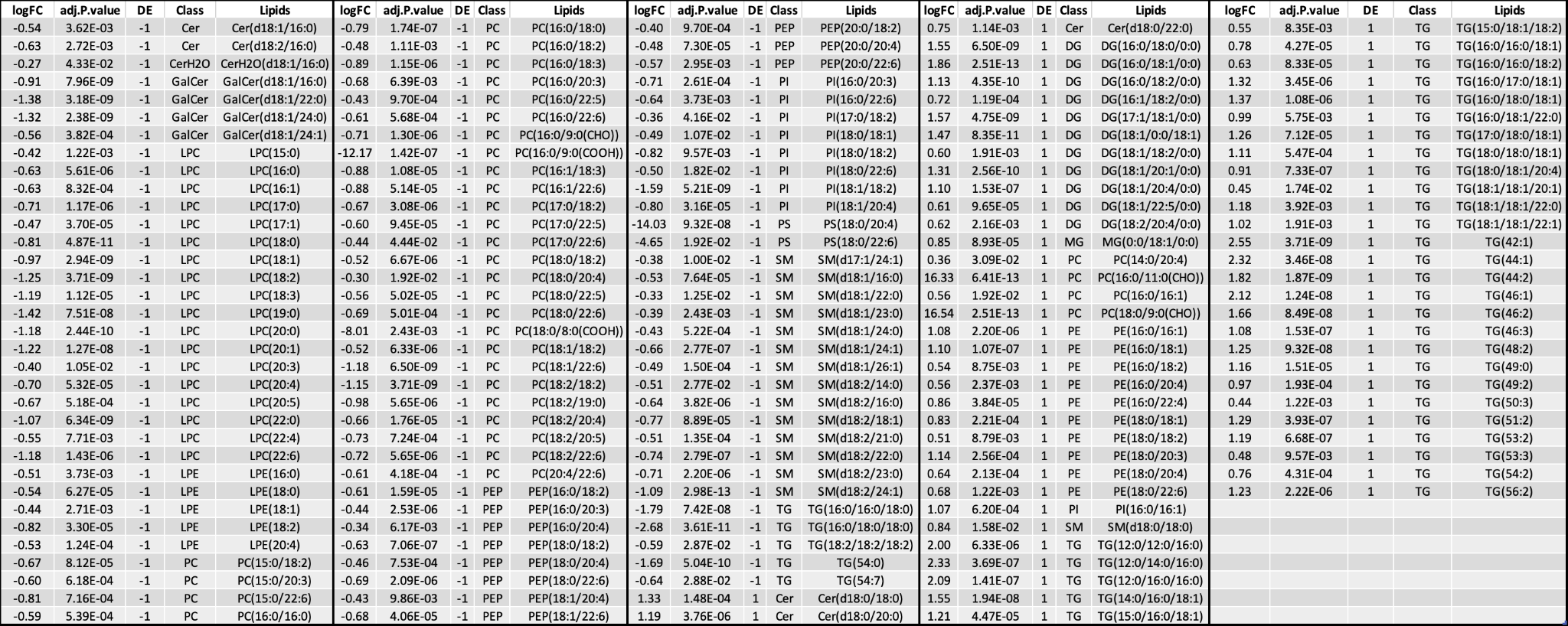


The heatmaps from ADViSELipidomics also depict these differences. The heatmaps in Figure 19 for DG species and Figure 20 for TG species show that most species are more abundant in case samples. The heatmaps of the remaining classes have a similar trend (data not shown).


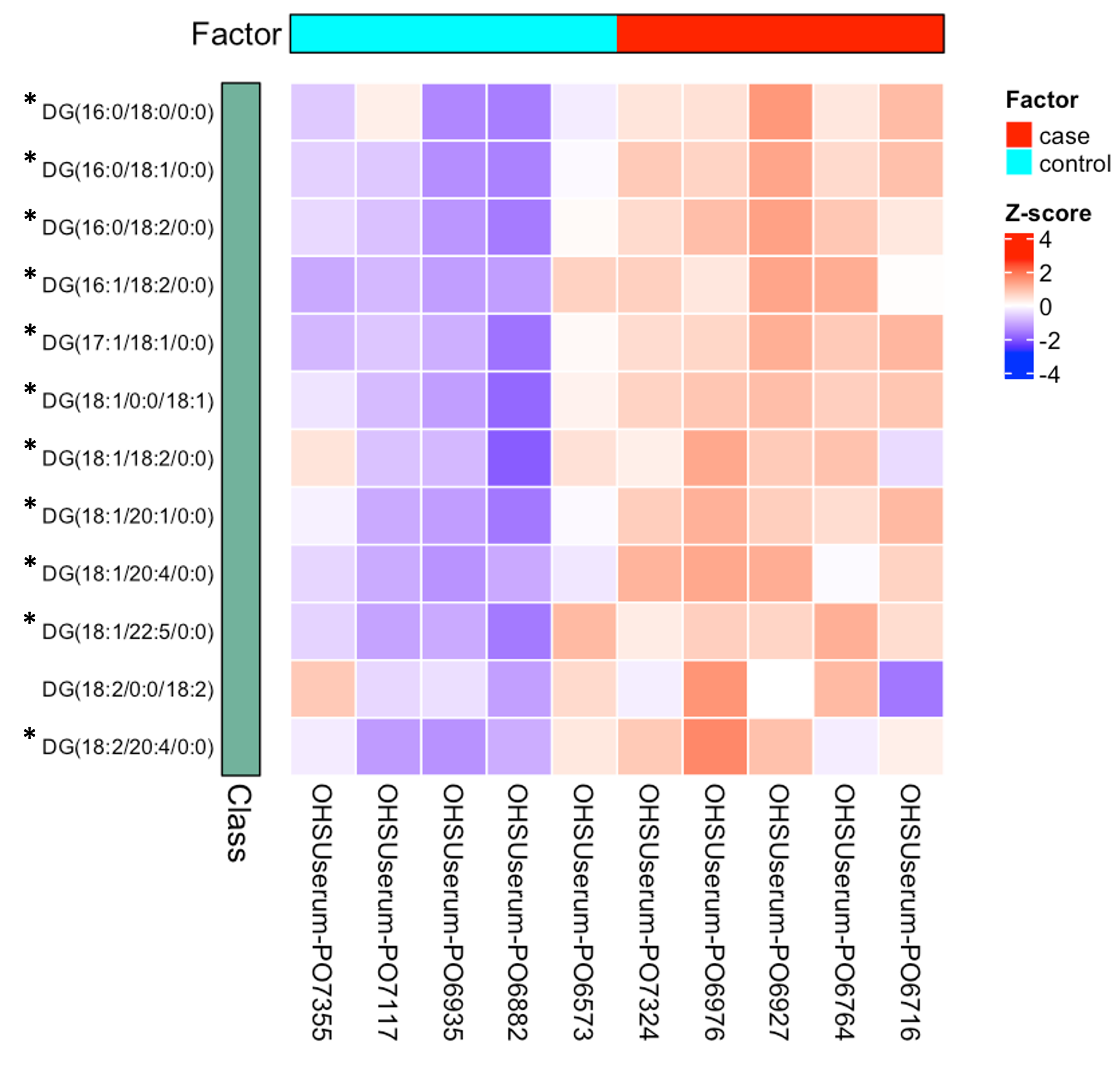


**Figure 19. Heatmap of DG species identified in serum control and serum case samples (Case Study Two).** The figure shows a clustering between serum case (red) and serum control (light blue) samples. Abundance values are converted to Z-score, with a gradient from high negative values (dark blue) to high positive values (dark red). DG species more abundant in serum case samples, statistically significant, are denoted with the asterisk (*).

***
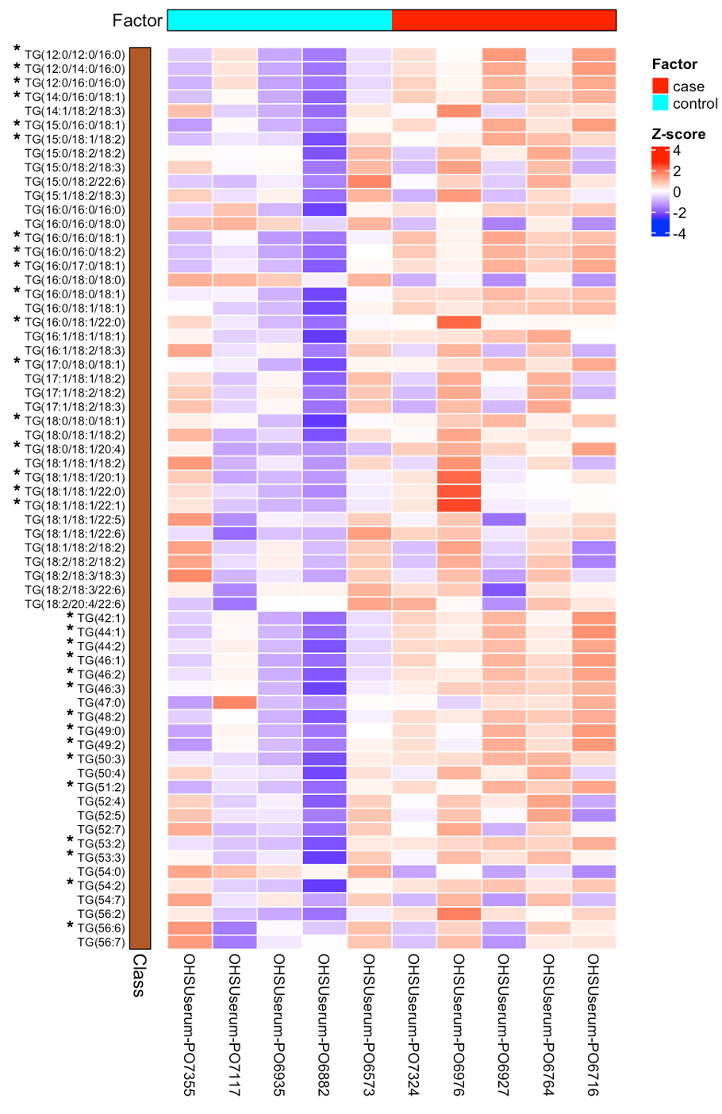
***

**Figure 20. Heatmap of TG species identified in serum control and serum case samples (Case Study Two).** The figure shows a clustering between serum case (red) and serum control (light blue) samples. Abundance values are converted to Z-score, with a gradient from high negative values (dark blue) to high positive values (dark red). TG species more abundant in serum case samples, statistically significant, are denoted with the asterisk (*).

**4. Analytic Details**

***4.1 Method Validation and Validation Study***

We validated the proposed LC-MS/MS method by following as closely as possible the guidelines prepared by the Food and Drug Administration (Food and Drug, FDA, 2018), the European Medicines Agency (EMA, 2019), and the articles with the methodology of validation (Della Corte et al., 2015; Iacuzzi et al., 2019; Wolrab et al., 2020). We performed the validation for each IS present in the IS Mix, and we used the IS Mix for calibration curves, to establish the lowest limit of quantification (LLOQ), and to spike into QC samples. We prepared QC samples by extracting lipids from primary monocyte derived-iDCs following the MTBE (Methyl Tert-Butyl Ether) extraction protocol detailed above, using the QC samples to evaluate the matrix factor and intra-day/inter-day accuracy and precision.

4.1.1 Chemicals and Reagents

We purchased HPLC-MS grade methanol from Merck (Darmstadt, Germany), and ammonium hydroxide solution (≥ 25% in water, eluent additive for LC-MS) and formic acid solution (98-100% in water, eluent additive for LC-MS) from Merck (St. Louis, MO, USA). We purchased the lipid standards detailed on Table 4 from Avanti Polar Lipids. For cell culture media: Roswell Park Memorial Institute 1640 medium (RPMI-1640) and fetal calf serum were all from Gibco (Thermo Fisher Scientific, Waltham, MA, USA); penicillin, streptomycin and L-glutamine were from Sigma-Aldrich, Inc.

**Table 4. List of ISs.** On the first column, the IUPAC name of each IS, on the second column the abbreviation of each lipid species, and on the third column the lipid species used to prepare IS Mix are marked with **✓**.

| Name | Sigla | IS Mix |
| --- | --- | --- |
| 1-pentadecanoyl-2-oleoyl(d_7_)-sn-glycero-3-phosphocholine | 15:0/18:1-d_7_ PC | **✓** |
| 1-pentadecanoyl-2-oleoyl(d_7_)-sn-glycero-3-phosphoethanolamine | 15:0/18:1-d_7_ PE | **✓** |
| 1-pentadecanoyl-2-oleoyl(d_7_)-sn-glycero-3-[phosphor-rac-(1’-glycerol)] | 15:0/18:1-d_7_ PG | **✓** |
| 1-pentadecanoyl-2-oleoyl(d_7_)-sn-glycero-3-phospho-L-serine | 15:0/18:1-d_7_ PG | **✓** |
| 1-pentadecanoyl-2-oleoyl(d_7_)-sn-glycero-3-phosphoinositol | 15:0/18:1-d_7_ PI |  |
| 1-heptadecanoyl-2-hydroxy-sn-glycero-3-phosphocholine | 17:0 Lyso PC | **✓** |
| 1-(10Z-heptadecenoyl)-sn-glycero-3-phosphoethanolamine | 17:1 Lyso PE | **✓** |
| 1-(10Z-heptadecenoyl)-sn-glycero-3-phospho-(1’-rac-glycerol) | 17:1 Lyso PG | **✓** |
| N-oleoyl(d_9_)-D-erythro-sphingosylphosphorylcholine | 18:1-d_9_ SM | **✓** |
| 1,3-dipentadecanoyl-2-oleyol(d_7_)-glycerol | 15:0/18:1-d_7_/15:0 TG | **✓** |
| 1-pentadecanoyl-2-oleyol(d_7_)-sn-glycerol | 15:0/18:1-d_7_ DG | **✓** |
| cholest-5-en-3β-yl heptadecanoate | 17:0 ChE | **✓** |
| N-heptadecanoyl-D-erythro-sphingosine | C17 Cer | **✓** |
| Sulfoquinovosyldiacylglycerol | SQDG |  |
| 1,2-dipalmitoyl-sn-glycero-3-O-4’-[N,N,N-trimethyl(d_9_)]-homoserine | DGTS-d_9_ |  |
| Monogalactosyldiacylglycerol (plant, hydrogenated) | MGDG |  |
| Digalactosyldiacylglycerol (plant, hydrogenated) | DGDG |  |

4.1.2 Standard Mixtures

We purchased most lipid standards in a chloroform solution at 1 mg/mL, used as stock solutions. We prepared a stock solution of 1 mg/mL in MeOH:CHCl_3_ (4:1, v/v) for the remaining lipid standards. From the stock solutions, by dilution in MeOH:CHCl_3_ (4:1, v/v), we prepared the IS Mix at 25 µg/mL in MeOH:CHCl_3_ (4:1, v/v).

4.1.3 Selectivity

To validate the selectivity of the analytical method, we injected QC samples non-spiked with IS Mix, followed by QC samples spiked with 3 µg/mL of IS Mix, in triplicate. As expected, we did not observe any IS peak for non-spiked QC samples.

4.1.4 External Calibration Curves

We prepared the calibration curves in triplicate in MeOH:CHCl_3_ (4:1, v/v) in the range 10 µg/mL – 0.1 ng/mL, in a total of 11 calibration curve points by a serial dilution of IS Mix. We evaluated the calibration curves either in HRESI (+) or HRESI (-). Furthermore, we built an external calibration curve for each lipid to evaluate possible differences in the ionization of the lipids. Finally, we evaluated the linearity of the analytical method using the r^2^ value, with an observed value of 0.995 or higher for all the classes, as reported in Table 5.

**Table 5. Calibration Curve Parameters.** In the first column is the list of the standards used to test the parameters of the calibration curves, the second column is the range of concentration, in ng/mL, in which each IS displayed linearity, the third column is the equation of each obtained calibration curve and on the fourth column the corresponding coefficient of determination (r^2^), on the last column is the lowest limit of quantification (LLOQ) for each IS measured as ng/mL.


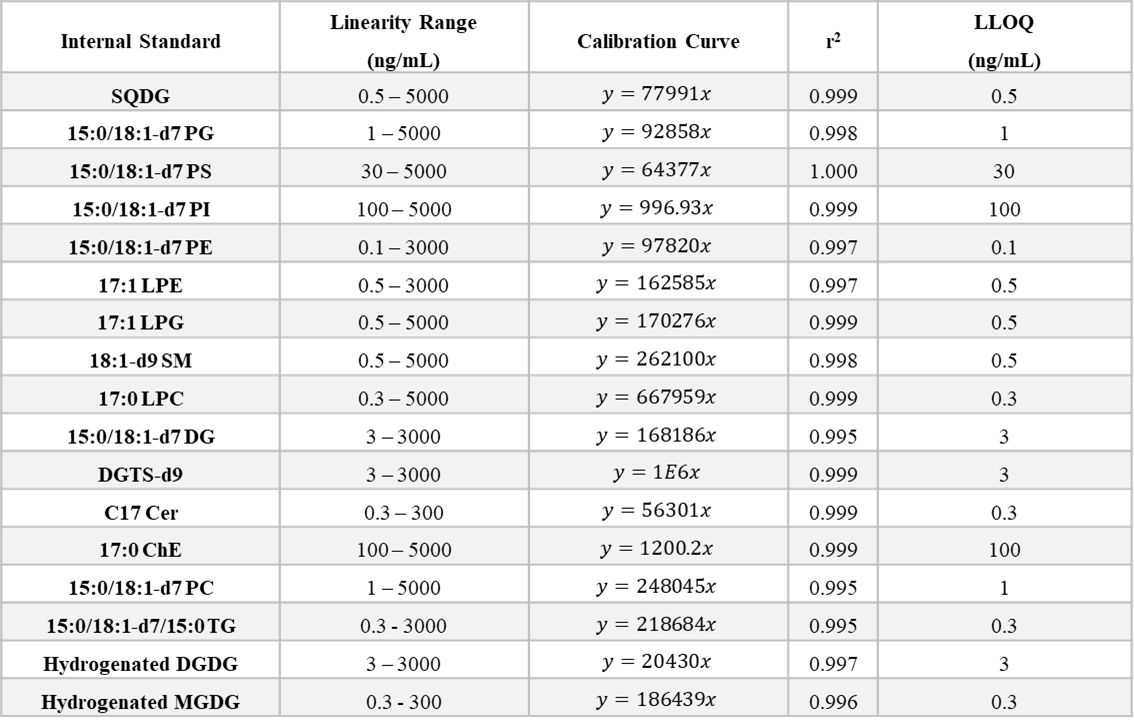


4.1.5 Lower Limit of Quantification

We calculated the LLOQ as the lowest concentration of analyte in a sample that could be reliably quantified, i.e., % CV < 20% and accuracy error ± 20%. Results are reported in Table 5.

4.1.6 Carry-over Effect

We evaluated the carry-over effect by injecting solvent blanks after injections of the most concentrated point of the calibration curve standard (5 μg/mL). After injecting two blanks, we did not observe any significant carry-over effect before proceeding with the injection of samples.

4.1.7 Matrix Effect

We determined the matrix effect by calculating the ratio of each IS spiked peak area after the extraction (QC samples) to the peak area in the absence of matrix (pure IS Mix). We evaluated matrix effect for all the 17 ISs used at 2 different concentration levels, namely at two times LLOQ and 3 µg/mL (high concentration level), in triplicate. The calculated % MF is reported in Table 6, with the corresponding % CV.

**Table 6. Matrix Effect.** The values in the table represent the matrix effect measured as matrix factor (%) and CV (%) at two concentration levels (2 x LLOQ and 3 µg/mL), in triplicate.


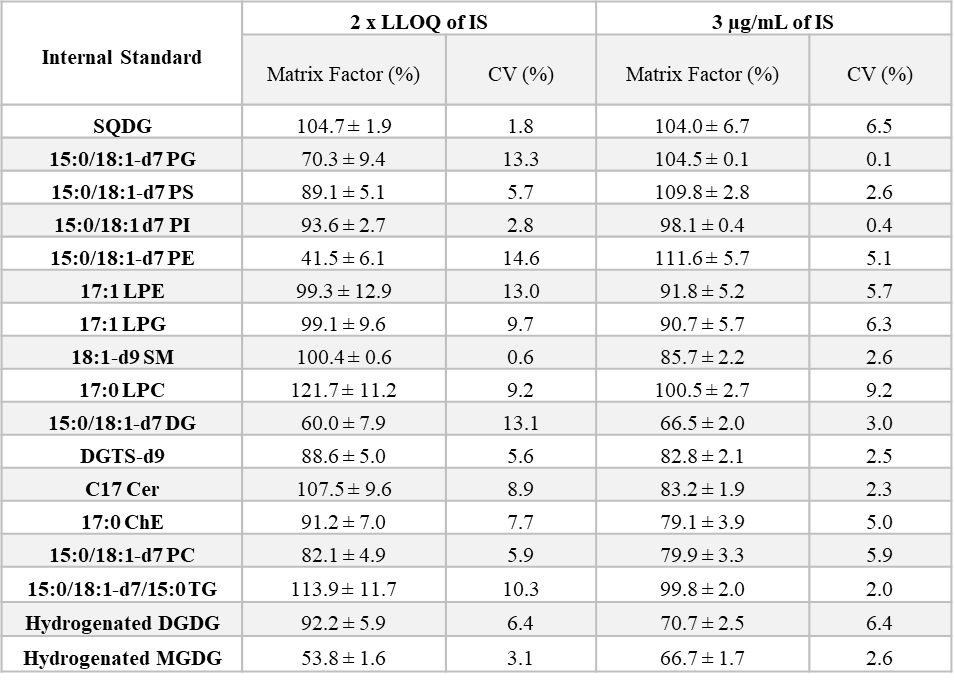


4.1.8 Accuracy and Precision

We accessed accuracy and precision on QC samples spiked with known IS amounts at two different concentrations. First, we spiked QC samples with IS Mix, in triplicate, at 300 ng/mL and 3 µg/mL. We calculated the concentrations of individual samples with the regression equation of a calibration curve measured at the beginning of the sequence.

For evaluating the accuracy, we subtracted the mean of the calculated concentration from the nominal concentration, dividing it by the nominal concentration for each level. We calculated the accuracy: a) for a single day by using a calibration curve injected at the beginning of the sequence for the determination of concentrations (within-run accuracy); b) for two different days by using a calibration curve measured at the beginning of the sequence for determination of concentration in two different days (between-run accuracy). Precision is expressed as % CV in Table 7. Overall, the analytical method displayed intra-run and between-run accuracy and precision at both concentration levels, with CV lower than 15% for most standards.

**Table 7. Within-run and between-run accuracy (Error %) and precision (CV %).** The values in the table represent within-run and in-between-run accuracy and precision (measured as Error % and CV %, respectively). We calculated accuracy and precision by spiking a mix containing the detailed ISs into QC samples, in triplicate, at 300 ng/mL and 3 µg/mL.


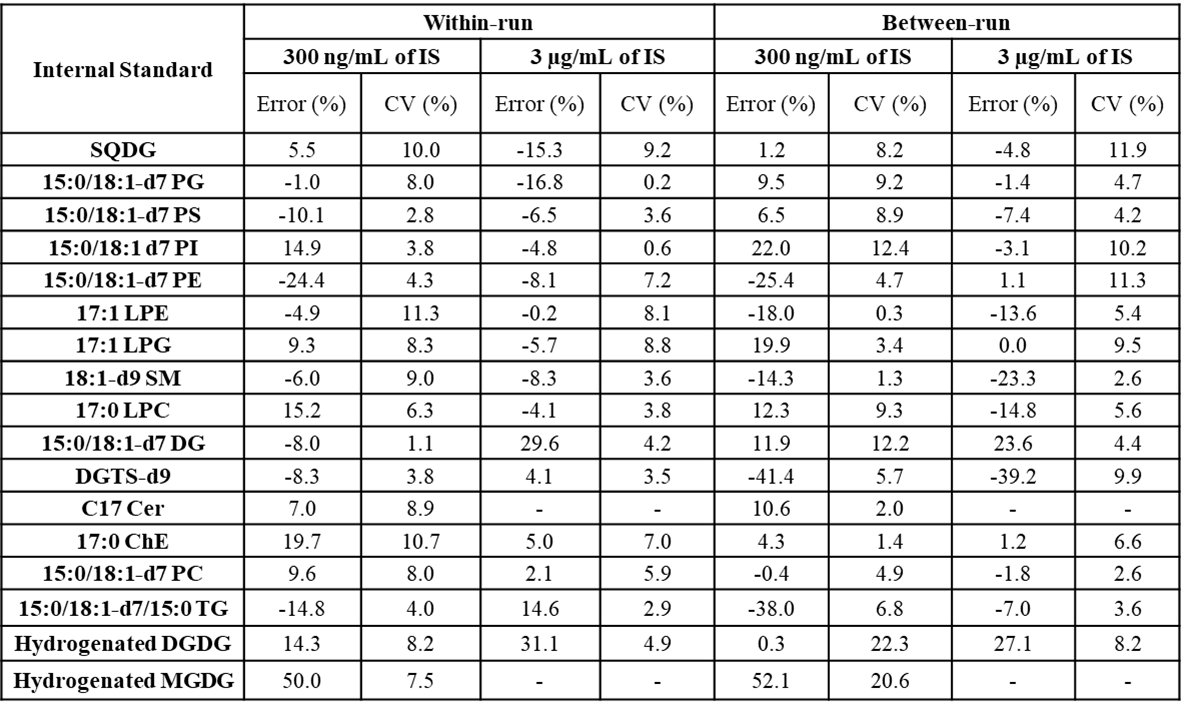


***4.2 Case Study One***

4.2.1 Human Monocyte Isolation

We obtained blood from healthy donors, and the Regional Ethics Committee for Clinical Experimentation of the Tuscany Region (Ethics Committee Register n. 14,914 of May 16, 2019) approved the protocol. We obtained peripheral blood mononuclear cells (PBMC) by Ficoll-Paque gradient density separation (GE Healthcare, Bio-Sciences AB, Uppsala, Sweden). According to the manufacturer's instructions, we isolated CD14+ monocytes from PBMC using anti-CD14 antibody-bearing magnetic microbeads (Miltenyi Biotec, Bergisch Gladbach, Germany). We assessed cell viability by trypan blue dye exclusion and found it to be > 95%. We determined monocyte purity microscopically after cytocentrifugation and differential staining with a modified Wright-Giemsa dye (Di-Quik; Medion Diagnostics, Duedingen, Switzerland). We used only preparations with > 95% of purity.

4.2.2 Immature Dendritic Cells Culture Conditions

We obtained iDCs incubating freshly isolated monocytes at 1x10^6^ cells per mL RPMI 1640 medium, supplemented with 10% fetal calf serum, 1% L-glutamine 2 mM, 1% penicillin and streptomycin, human IL-4 (5 ng/ mL) and human GM-CSF (100 ng/ mL) for five days.

4.2.3 Lipid Extraction

Following the harvesting of cells, we extracted the lipids based on a modified MTBE method (Nuzzo et al., 2018). We spiked IS Mix (Table 4) into the pellets of innate immune cells, followed by 900 µL of LC-MS grade MeOH. After a vigorous vortex, we added 3 mL MTBE and left the extraction shaking at room temperature for 1h. Upon adding 750 µL of milliQ.H2O and shaking at room temperature for another 10 min, we centrifuged the samples at 3000 rpm for 10 min at 4 ºC. We collected the upper organic phase to a glass vial, and we added 1 mL of MTBE to the remaining aqueous phase. Upon shaking at room temperature for 10 min, we centrifuged the samples and combined the upper phase with the previous one. Finally, we dried the lipid extracts under a nitrogen stream and dissolved them in MeOH:CHCl_3_ (4:1, v/v) before further LC-MS analysis.

4.2.4 UHPLC-HRESIMS-MS/MS

We performed chromatographic separations on Infinity 1290 UHPLC System (Agilent Technologies, Santa Clara, CA, USA), equipped with a Kinetex Biphenyl 2.6 μm, 150 x 2.1 mm column (Phenomenex, Castel Maggiore, Bologna, Italy) at 28 ºC. Eluent A: water and eluent B: MeOH with 0.005% NH_4_OH adjusted to pH 8 with Acetic Acid. The elution program consisted of a gradient from 60% to 80% of B in 2 minutes, then 100% of B in 15 min, holding at 100% B for 15 min and returning to 60% of B at 33 min followed by an equilibration step of 7 min at 60% B prior to the following injection. We carried MS and MS/MS analyses out on a Q-Exactive Hybrid Quadrupole – Orbitrap mass spectrometer (Thermo Scientific, San Jose, CA, USA) equipped with a HESI source. Source parameters were as follows: spray voltage positive polarity 3.2 kV, negative polarity 3.0 kV, capillary temperature 320 ºC, S-lens RF level 55, Auxiliary gas temperature 350 ºC, sheath gas flow rate 60, auxiliary gas flow rate 35. We acquired full MS scans over the range m/z 150 – 1800, with a mass resolution of 70,000. The target value (AGC) was 1e6, and the maximum allowed accumulation time (IT) was 100 ms. We used dependent MS/MS (ddMS2) analysis for the data, a Top10 method. We selected the ten most intense peaks for fragmentation with stepped normalized energy of 25/28/35 in positive ionization mode and 20/40 in negative ionization mode. AGC was 1e5 with IT 75 ms and 17,500 mass resolution. The injection volume was 10 μL. We submitted the LC-MS/MS spectra to LipidSearch 4.2.29 (Thermo Scientific, San Jose, CA, USA), and the search criteria were as follows: product search; parent m/z tolerance 5 ppm; product m/z tolerance 5 ppm; Quantitation: m/z tolerance 5 ppm; RT tolerance 0.5 min. Lipids were identified against the general and labeled databases. We identified lipids as adduct [M+H]^+^, [M+Na]^+^, [M+NH_4_]^+^ in positive mode, and as [M-H]^-^ in negative mode, searching the following lipid classes: Cer, LPC, LPE, PC, PE, PG, PS, SM, DG, ChE and TG.

**5. Acknowledgements**

The authors thank Giuliana d’Ippolito and Emiliano Manzo for the support on the improvement of the LC-MS/MS method and the organization of the experiments within the ADViSE project.

**6. Funding**

This work was supported by the project “*Antitumor Drugs and Vaccines from the Sea (ADViSE)*” project (CUP B43D18000240007–SURF 17061BP000000011) funded by POR Campania FESR 2014-2020 “Technology Platform for Therapeutic Strategies against Cancer” - Action 1.2.1 and 1.2.2.

**7. References**

Chang,W. *et al.* (2021) shiny: Web Application Framework for R. R package version 1.7.1.

Coillard,A. *et al.* (2019) In vivo Differentiation of Human Monocytes. *Front. Immunol.* **10**:1907.

Colombo,S., *et al*. (2018) Phospholipidome of endothelial cells shows a different adaptation response upon oxidative, glycative and lipoxidative stress. *Sci. Rep.*, **8**(1), 12365.

Cutignano,A., *et al*. (2016) Profiling of complex lipids in marine microalgae by UHPLC/tandem mass spectrometry. *Algal Res*., **17**, 348–58.

Della Corte,A. *et al.* (2015) A rapid LC-MS/MS method for quantitative profiling of fatty acids, sterols, glycerolipids, glycerophospholipids and sphingolipids in grapes. *Talanta*, **140**, 52-61.

EMA/CHMP/192217/2009 Rev. 1 Corr. 2** EMA. (2009) Guideline on bioanalytical method validation. http://www.ema.europa.eu/en/documents/scientific-guidelines/guideline-bioanalytical-method-validation_en.pdf.

Fay,C. *et al.* (2021) golem: A Framework for Robust Shiny Applications. R package version 0.3.1.

Food and Drug Administration UD of H and HSC for DE and R and C. (2018) Bioanalytical Method Validation, Guidance for Industry. http://www.fda.gov/cder/guidance/4252fnl.htm.

Grolemund,G. *et al.* (2017) R for Data Science. O’Reilly Media.

Gu,Z. *et al.* (2016) Complex heatmaps reveal patterns and correlations in multidimensional genomic data. *Bioinformatics*, **32**(18), 2847-2849.

Huber,W. *et al.* (2015) Orchestrating high-throughput genomic analysis with Bioconductor. *Nat. Methods*, **12**(2), 115-121.

Iacuzzi,V. *et al.* (2019) Development and validation of LC-MS/MS method for imatinib and norimatinib monitoring by finger-prick DBS in gastrointestinal stromal tumor patients. *PLoS One*, **14**(11):e0225225.

Leek,J.T. *et al.* (2012) The sva package for removing batch effects and other unwanted variation in high-throughput experiments. *Bioinformatics*, **28**(6), 882-883.

Le Naour,F., et al. (2001) Profiling changes in gene expression during differentiation and maturation of monocyte-derived dendritic cells using both oligonucleotide microarrays and proteomics. *J. Biol. Chem.*, **276**(21), 17920-31.

Lloyd,G.R. *et al.* (2021) metabolomicsWorkbenchR: Metabolomics Workbench in R. R package version 1.4.0.

Łuczaj,W., *et al*. (2016) Phospholipidomic Analysis Reveals Changes in Sphingomyelin and Lysophosphatidylcholine Profiles in Plasma from Patients with Neuroborreliosis. *Lipids*, **52**(1), 93–8.

Luczaj,W., *et al*. (2018) Plasma lipidomic profile signature of rheumatoid arthritis versus Lyme arthritis patients. *Arch. Biochem. Biophys*., **654**, 105–14.

Lühr,J.J., et al. (2020) Maturation of Monocyte-Derived DCs Leads to Increased Cellular Stiffness, Higher Membrane Fluidity, and Changed Lipid Composition. *Frontiers in immunology*, ***11***, 590121.

Kassambara,A. *et al.* (2020) factoextra: Extract and Visualize the Results of Multivariate Data Analyses. R package version 1.0.7.

Korotkevich,G. *et al.* (2019) Fast gene set enrichment analysis. *bioRxiv*, **060012**.

Kowarik,A. *et al.* (2016) Imputation with the R Package VIM. *Journal of Statistical Software*, **74**(7), 1–16.

Kyle,J.E. *et al.* (2017) LIQUID: an-open source software for identifying lipids in LC-MS/MS-based lipidomics data. *Bioinformatics*, **33**(11), 1744-1746.

Maechler,M. *et al.* (2021) cluster: Cluster Analysis Basics and Extensions. R package version 2.1.2.

Mohamed,A. *et al.* (2020) lipidr: A Software Tool for Data Mining and Analysis of Lipidomics Datasets. *J. Proteome Res.*, **19**(7), 2890-2897.

Morgan,M. *et al.* (2021) SummarizedExperiment: SummarizedExperiment container. R package version 1.24.0.

Nuzzo,G. *et al.* (2018) UPLC⁻MS/MS Identification of Sterol Sulfates in Marine Diatoms. *Mar. Drugs*, **17**(1):10.

Ocaña-Morgner,C., *et al*. Tolerogenic versus Immunogenic Lipidomic Profiles of CD11c + Immune Cells and Control of Immunogenic Dendritic Cell Ceramide Dynamics. *J. Immunol.*, **198**(11),4360–72.

Pakiet,A., *et al*. (2021) Alterations in complex lipids in tumor tissue of patients with colorectal cancer. *Lipids Health Dis.*, **20**(1), 85.

Patterson,A.D., *et al*. (2011) Aberrant Lipid Metabolism in Hepatocellular Carcinoma Revealed by Plasma Metabolomics and Lipid Profiling. Cancer Res., **71**(21), 6590 LP – 6600.

Puri,P., *et al*. (2009) The plasma lipidomic signature of nonalcoholic steatohepatitis. *Hepatology,* **50**(6), 1827–38.

Ritchie,M.E. *et al.* (2015) limma powers differential expression analyses for RNA-sequencing and microarray studies. *Nucleic Acids Research*, **43**(7):e47.

Rohart,F. *et al.* (2017) mixOmics: An R package for ‘omics feature selection and multiple data integration. *PLoS Comput. Biol.*, **13**(11):e1005752.

Santinha,D.R., *et al*. (2012) Profiling changes triggered during maturation of dendritic cells: A lipidomic approach. *Anal Bioanal. Chem*., **403**(2), 457–71.

Satomi,Y. *et al.* (2017) One-step lipid extraction for plasma lipidomics analysis by liquid chromatography mass spectrometry. *J. Chromatogr. B. Analyt. Technol. Biomed. Life Sci.*, **1063**, 93-100.

Sievert,C. (2020) Interactive Web-Based Data Visualization with R, plotly, and shiny. *Chapman and Hall/CRC Florida*.

Tang-Huau,T.L., Segura,E., (2019) Human in vivo-differentiated monocyte-derived dendritic cells. Semin. *Cell. Dev. Biol*., **86**, 44-49.

Tiwari-Heckler,S., *et al*. (2018) Circulating Phospholipid Patterns in NAFLD Patients Associated with a Combination of Metabolic Risk Factors. *Nutrients,* ***10****(5), 649.*

Wolrab,D. *et al.* (2020) Validation of lipidomic analysis of human plasma and serum by supercritical fluid chromatography-mass spectrometry and hydrophilic interaction liquid chromatography-mass spectrometry. *Anal. Bioanal. Chem.*, **412**(10), 2375-2388.

Zhao,Y.Y., *et al.* (2015) Lipidomics: Novel insight into the biochemical mechanism of lipid metabolism and dysregulation-associated disease. *Chem. Biol. Interact.*, **240**, 220-238.
